# Are Full-Night Samplings Necessary? Unraveling the Hourly Structure and Climatic Responses of Three Moth Groups in a Brazilian Pampa Grassland

**DOI:** 10.1007/s13744-026-01394-7

**Published:** 2026-04-29

**Authors:** Matheus Eduardo Schwantes, Isabela Andrade Bahima, Viviane Gianluppi Ferro

**Affiliations:** 1 Programa de Pós Graduação Em Biologia Animal, Instituto de Biociências, Univ Federal Do Rio Grande Do Sul, Porto Alegre, Brazil; 2 Curso de Ciências Biológicas, Instituto de Biociências, Univ Federal Do Rio Grande Do Sul, Porto Alegre, Brazil; 3 Depto de Zoologia, Instituto de Biociências, Univ Federal Do Rio Grande Do Sul, Porto Alegre, Brazil

**Keywords:** Arctiinae, Natural history, Neotropical Lepidoptera, Saturniidae, Sphingidae

## Abstract

The daily activity of a species can be affected by several biotic and abiotic factors. Since the activity patterns and flight times of Neotropical moths are both poorly understood, our goal was to record and analyze the flight times of moths from the Pampa biome, in the extreme south of Brazil, as well as its responses to climatic factors. We recorded hours of activity and investigated the influence of four climatic variables on the activity patterns of Arctiinae (Erebidae), Sphingidae, and Saturniidae. Data analysis showed that different species have different activity periods, and the abundance and richness of each taxon has its own patterns. Arctiinae had a higher abundance in the early evening, although it did not show a richness concentration at any hour. Saturniidae and Sphingidae had both abundance and richness concentrated in the middle of the night. Climatic variables related to abundance and richness differed between taxa and species. We provide evidence of possible niche partitioning and differential physiological adaptations between taxa, as well as differences in flight time strategy between potential competing species. Our study highlights the necessity of full-night sampling for optimal faunal inventories.

**Supplementary Information:**

The online version contains supplementary material available at 10.1007/s13744-026-01394-7.

## Introduction

An organism’s daily activity is driven by several factors, such as climate, food availability, competition, and predation. Studies on the daily activity of species are relevant for answering both technical questions (e.g., determining optimal sampling times for a taxon) and scientific questions (e.g., understanding temporal niche partitioning and co-occurrence patterns) (Moreno et al. 2021). Although Insecta is the most diverse group of animals on the planet, studies focused on its daily activities are still scarce (Scherrer et al. 2013; Teston and Corseuil 2004). Within insects, Lepidoptera is considered to be a megadiverse order. In Brazil, there are almost 26000 recorded species (Carneiro et al. 2024). In the Brazilian Pampa, Lepidoptera currently has 747 recorded species, mostly butterflies (Andrade et al. 2023). However, this proportion is due to a higher taxonomic understanding and sampling effort of Papilionoidea, and does not necessarily correspond to the real Lepidoptera diversity of the region. Historically neglected, the Pampa biome has received attention over the last few years due to its great variety of grassland formations and its little-known biodiversity, which is highly threatened by the expansion of agriculture and forestry (Souza et al. 2020).

In this study, we selected three distinct taxa of Lepidoptera, aiming to visualize how different species behave in the same environment. By analyzing distinct groups, we can compare how different sizes, morphologies, and physiologies respond to climate in different moments of the night. Furthermore, we can infer whether potentially competing species are mutually exclusive or if they can coexist. The three following taxa were selected: Arctiinae (Erebidae), Sphingidae, and Saturniidae. These groups are highly diverse in the Neotropical realm and are composed mainly of nocturnal species (Carneiro et al. 2024; Heppner 1991; Kawahara et al. 2018). Despite the subfamily status, Arctiinae is the most diverse one, with approximately 11000 species worldwide and 1400 in Brazil (Ferro and Diniz 2010; Jacobson and Weller 2002). Next we have Saturniidae, with approximately 3500 species known worldwide and about 480 occuring in Brazil (Carneiro et al. 2024; Nunes 2006). At last, Sphingidae is known for more than 1600 species worldwide, including 196 in Brazil (Carneiro et al. 2024; Corrêa 2017; Haxaire and Mielke 2019; Specht et al. 2008a).

While Saturniidae and Sphingidae adults are generally medium- or large-sized moths, Arctiinae adults have medium to small sizes, being extremely diminute in some cases (e.g., lichen moths—Lithosiini). Both Arctiinae and Sphingidae adults are nectar feeders and potential pollinators, and many Sphingidae have direct associations with sphingophilous plants, which are pollinated only by sphingids and are characterized by elongated flowering tubes or a dense array of stamens (Amorim 2008). This sort of association causes several species of sphingidae to have very elongated proboscids. In contrast, adult Saturniidae are characterized by lacking or having nonfunctional mouthparts (Costa Lima 1950). Therefore, adults do not feed and survive on energy stored in the body during larval stage (Janzen 1984).

Saturniidae and Arctiinae are highly distinct in size, but both comprehend species with various morphological defense strategies. Saturniids can have vibrant colors or camouflage patterns, and many have wings with ocelli or translucent areas. Many Arctiinae species are wasp mimics or display aposematic colors that signal chemical defenses, those being generally sequestered from their hostplants (Molina and Di Mare 2017; Zaspel et al. 2014). Despite also being medium- to large-sized moths, Saturniidae body and wing proportions have many distinctions when compared to Sphingidae. In sphingids, the wing area is relatively small compared to body size, a morphological trait that allows a vigorous and well-directed flight (Carneiro et al. 2024; Machado 2014). In Saturniidae, the body is much smaller compared to the wing area (Carneiro et al. 2024).

Many factors can determine a species’ hour of activity. It is known that species that compete for a resource can either mutually exclude each other from habitats or coexist, with one form of coexistence being the utilization of the same resource in a slightly different way (Begon et al. 2007). In this case, having different foraging times could be a way for species to share floral resources without direct competition. Similarly, the presence of predators can be a determining factor in a species’ activity. Arctiinae and Sphingidae both comprise species with acoustic defenses against predators, either through eardrums for acoustic vigilance or through sound-producing mechanisms that hinder or inhibit predation by bats (Barber and Kawahara 2013; Dowdy and Conner 2019; Dunning et al. 1992; Göpfert et al. 2002; Kawahara et al. 2019). Saturniids, on the other hand, do not have eardrums or sound production structures, and rely on a variety of morphological adaptations on body and wings for survival (Barber et al. 2015; Ntelezos et al. 2017; Zeng et al. 2011).

In addition to biotic factors such as competition and predation, abiotic factors (such as temperature, relative humidity, and precipitation) directly influence moth biology. In Arctiinae, there is a general tendency of a decreasing number of generations with the increase of latitude, a pattern also recorded for several species of Sphingidae (Wagner 2009; Wiker et al. 2010). In Saturniidae, different species in the same region may present different numbers of generations per year (Janzen 1987; Nath et al. 2021; Tikader et al. 2013). As poikilothermic animals, insects and their development, survival, and distributions in different scales are strongly influenced by temperature (Régnière et al. 2012). Nevertheless, the effects of temperature and climate in the daily activity of Neotropical moths remain vastly understudied.

The present study aimed to (1) record the temporal activity patterns of different moth groups (lineages) and species throughout the night in a Pampa region of southern Brazil and (2) analyze the influence of key climatic factors (temperature, relative humidity, wind speed, and precipitation) on these patterns. In addition to registering which species occur in the area, we sought to (i) understand how species composition changes throughout the night, (ii) determine whether different taxonomic groups respond distinctly to climatic factors, (iii) evaluate the relationship between each group’s abundance/richness and climatic variables, and (iv) establish whether full-night sampling is necessary to obtain a representative community sample. We tested three hypotheses. The first is that different taxa would have different abundance/richness concentrations throughout the night. The second hypothesis is that smaller species (Arctiinae) would be concentrated in the early hours of the night, while larger species (Sphingidae and Saturniidae) would have a greater permanence capacity in the late night, given the higher heat retention capacity of moths with bigger bodies (Bartholomew and Heinrich 1973). The third is that climatic variables such as rain and wind would be more relevant for small species, since the impact of those is greater on smaller-sized structures (Ortega-Jimenez and Dudley 2012). This work is the first to analyze the hours of activity in a moth community of the Pampa, a Brazilian biome with a reduced area, but highly diverse when considering its extension (Andrade et al. 2023).

## Material and Methods

### Study Area

**Fig. 1:**
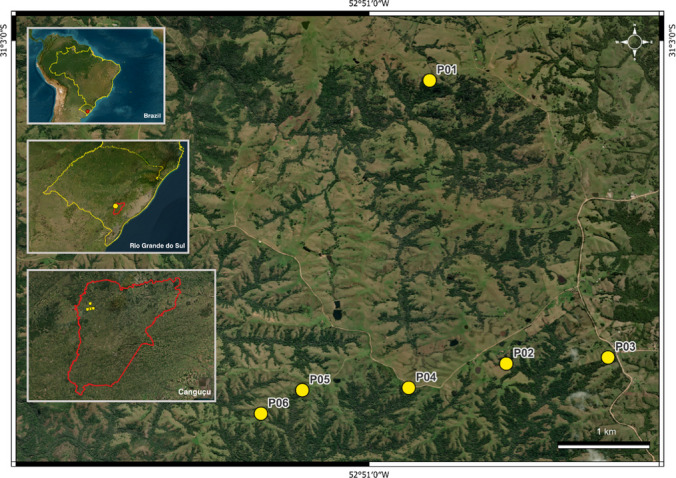
Location of the study area, in the municipality of Canguçu (marked in red), in the state of Rio Grande do Sul, Brazil, with the six sampling points highlighted in yellow

The study was conducted on a series of private properties in the “Coxilha do Fogo” locality, in the municipality of Canguçu, Rio Grande do Sul, Brazil (Fig. 1). The region is part of the Pampa biome (IBGE 2024). The relief is undulating, with small hills and mountains, mostly covered by savannah-like grassland formations of true grasses and herbaceous plants (Boldrini 2009; Minervini et al. 2023; Rambo 1956). Denser, smaller forests are present in the narrow valleys between hills and in small patches across the landscape (Boldrini 1997; Minervini et al. 2023). The altitude ranges from 200 to 500 m, and the climate is temperate in higher altitudes and subtropical in the lower regions (Boldrini 1997). The average temperature of the warmest month (January) is 24 °C, and in the coldest month (June) is 12.5°C (Girardi-Deiro et al. 2002). Precipitation is relatively well distributed throughout the year, with a monthly average of 125 mm; however, drought periods are common between December and March, while frosts occur between April and October (Caporal and Boldrini 2007; Girardi-Deiro et al. 2002). The soil is gravelly and shallow (Boldrini 1997). The main economic activity in the region is livestock farming of cattle and sheep (Bilenca and Miñarro 2004; Girardi-Deiro et al. 1994). A common practice is the slash-and-burn of shrub vegetation in order to expand the livestock grazing area (Boldrini 1997).

### Sampling

The samplings occurred in summer (December 13–19, 2023) during the new moon phase, as light trap efficiency is higher in darker nights. Six sampling points were selected, all located on forest edges (Fig. 1). Trampling and grazing by animals (primarily sheep, but also goats, cattle and horses) were present through the whole area. To ensure an independent sampling, the minimum distance between points was 500 m. This distance is equivalent to 2.5 times the radius of attraction of the light source used in this study (Muirhead-Thompson 1991). Each point was sampled for one night, from dusk to dawn. The night was divided into 1-h periods, starting at 20:00 and ending at 04:59.

Moths were sampled using an active method (white sheet). The light source was a 250 W mixed-light mercury lamp connected to an electric generator. The sheet was 2.40 m wide by 1.80 m high and hung at a height of 2 m. Adult moths belonging to the Arctiinae, Sphingidae, and Saturniidae were captured when they landed on the sheet or in surrounding vegetation (approximately 2 m radius). Individuals were euthanized with a kill jar containing ethyl ether and stored in envelopes with corresponding sampling hours. Subsequently, specimens were pinned, labeled, and identified through morphological characters of the wings, body, and appendages with the help of the bibliography (Camargo et al. 2018; Dowdy et al. 2020; Haxaire and Mielke 2019; Kitching 2025; Martin et al. 2011; Nunes 2006; Pinheiro and Gaal-Haszler 2015; Specht et al. 2008a, 2008b; Vincent and Laguerre 2014) and through comparison with specimens from the UFRGS Lepidoptera Collection.

Climate data were recorded in situ, 30 min after the start of each hour using a digital thermo hygrometer and a portable weather station (Table S. 1). Three continuous variables were recorded: temperature, relative humidity, and wind speed, the last one being the average between the maximum and minimum values recorded 30 min after the start of each hour. Precipitation was recorded categorically as null, light, medium, or heavy.

### Data Analysis

The number of species in the study area was calculated using the Chao 2 and Jackknife 1 richness estimators. We performed circular analyses with the Rayleigh test (Zar 1996) for the Total set of moth species and separately for Arctiinae, Sphingidae, and Saturniidae using the Oriana software (Kovach 2013), aiming to visualize possible concentrations of abundance and richness in any specific hour.

To check relations between abundance and richness with climatic variables, Generalized Linear Models (GLMs) were performed in R software version 4.4.2 (R Core Team 2024) with the help of *ggplot2*, *stats*, *MASS*, *DHARMa*, and *stargazer* packages (Hartig 2024; Hlavac 2022; R Core Team 2024; Venables and Ripley 2002; Wickham 2016). GLMs were performed for all species (Total), for each taxon separately (Arctiinae, Sphingidae, and Saturniidae), and also for the 13 species with the highest abundance and/or occurrence in every hour of the night. Preliminary models were initially tested with two different statistical distributions (Poisson and Negative Binomial) and compared with the Akaike Information Criterion (AIC) to determine which model best suited the data. After choosing the distribution structure, models were built with the response variables *temperature*, *humidity*, *wind*, *precipitation*, and *hours after sunset*, subsequently using ANOVA to remove non-significant variables. Negative binomial models were supported by ANOVA for most of the analyses performed; Poisson-distributed models were better suited in analyses where the data distribution peaked abruptly at specific hours.

The morphospecies *Lonomia* sp. was removed from the “Total” and “Saturniidae” GLM analysis since throughout its occurrence period, it was indifferent to precipitation and other climatic factors. Due to its apparent resilience to unfavorable climate, models built with the inclusion of *Lonomia* sp. had a high interference level and were not consistent with the general patterns observed in the other Saturniidae species.

## Results

### Abundance and Richness of Moths in the Coxilha do Fogo

We sampled a total of 1382 adult moths. Of these, 1318 were arctiines (95.4%), 39 sphingids (2.8%), and 25 saturniids (1.8%) (Table S. 2). Total species richness was 94 (74 species of Arctiinae, 12 of Sphingidae, and 8 of Saturniidae) (Table S. 2). Fourteen morphospecies were identified only to genus level, one was identified to subtribe level, and one to tribe level. The richness found represents approximately 95% of the estimated richness for the area (Chao 2 = 100.5; Jackknife 1 = 97.6). In total, 24.5% of the species were represented by only one individual (23 singletons) and 69 species (73.4%) by 10 or fewer individuals (Fig. S. 1). The five most abundant species were *Heliactinidia nigrilinea* (Walker, 1856) (235 individuals), *Eurata hilaris* Zerny, 1937 (99 individuals), *Bertholdia almeidai* Travassos, 1950 (85 individuals), *Ctenucha rubriceps* Walker, 1854 (83 individuals), and *Lamprostola pascuala* (Schaus, 1896) (69 individuals), all belonging to the Arctiinae (Table S. 2).

Moth abundance and richness varied between nights. As sampling points were essentially identical (despite different locations), the variations were driven by the climatic conditions of each night. The nights with the highest and lowest abundance had 551 and 69 individuals, respectively. The nights with the highest and lowest richness had 71 and 28 species, respectively. We detected a significant and positive relationship between abundance and richness (*R*^2^ = 0.99, *F* = 959.80, *p* < 0.0001).

### Community Structure Throughout the Night

**Fig. 2:**
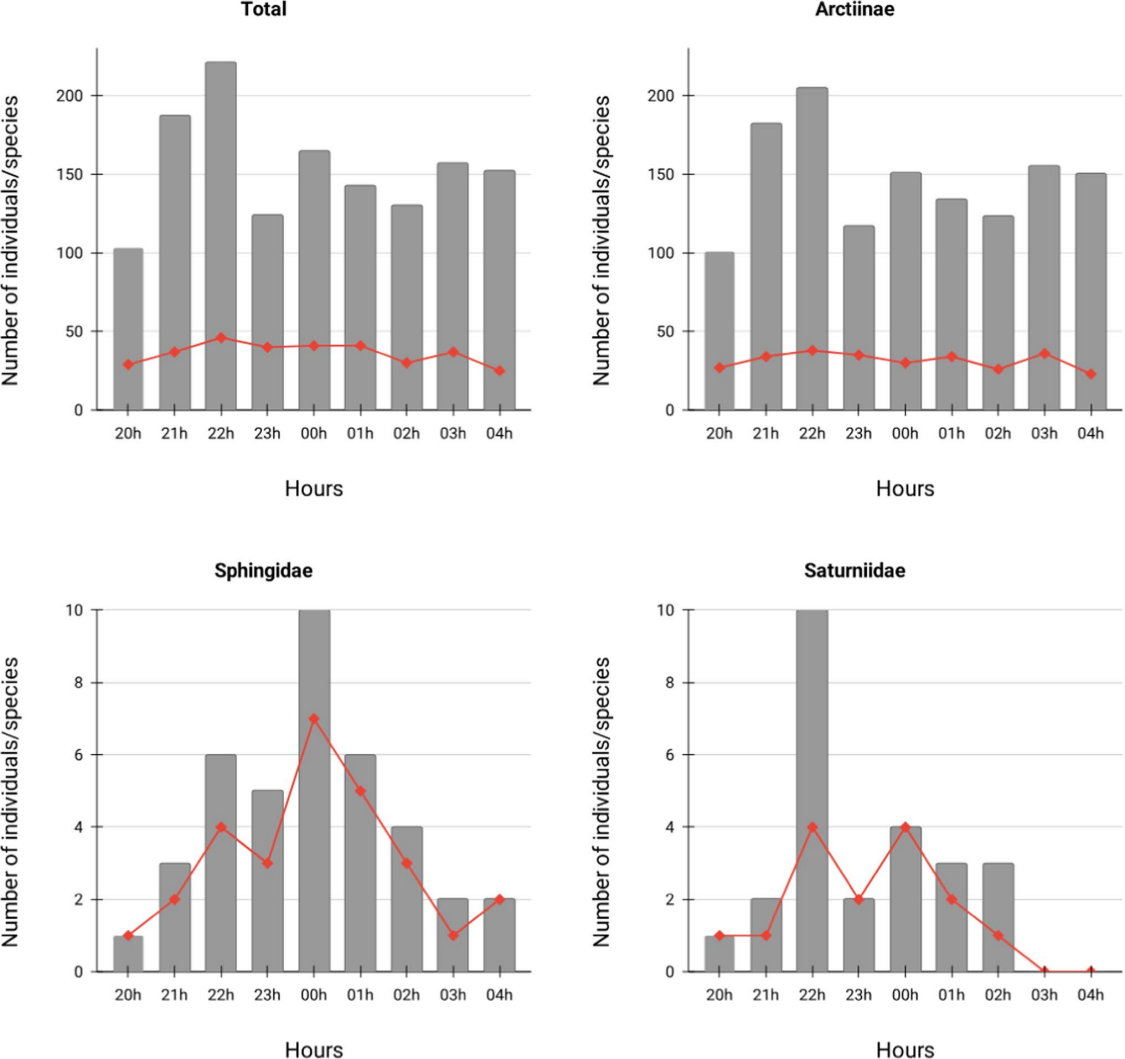
Abundance (gray bars) and richness (red lines) of moths throughout the night

For the Total sample, 22 h was the time of higher abundance (221 individuals) and richness (46 species); the lowest abundance was at 20 h (103 individuals) and the lowest richness at 04 h (25 species) (Fig. 2). For Arctiinae, the time of higher abundance and richness was at 22 h (205 individuals, 38 species); the lowest abundance was at 20 h (101 individuals) and the lowest richness was at 04 h (23 species). In Sphingidae, the highest abundance (10 individuals) and richness (7 species) occurred at midnight; the hour of least abundance was at 20 h (1 individual) and the times of lowest richness were at 20 h and 03 h (both with only one species each). In Saturniidae, the highest abundance was at 22 h (10 individuals) and the highest richness occurred at 22 h and 00 h (both with 4 species). Saturniids were completely absent in the final hours of the night (03–04 h).

Total abundance peaked in the early hours after dusk, decreasing and remaining relatively constant until the end of the night (Fig. 2). Circular analysis showed that abundance was concentrated over time for the Total sample, for Arctiinae, Sphingidae, and Saturniidae (Table 1, Fig. S. 3). The 22-h time period was characterized by the highest richness for both the total sample and Arctiinae (46 and 38, respectively; Fig. 2). However, the richness distribution was dispersed throughout the night for Arctiinae (*r* = 0.06, *Z* = 1.025, *p* = 0.359) (Table 1), despite the significant concentration at 23 h in the Total sample analysis. Saturniidae and Sphingidae had richnesses concentrated in the middle of the night period (23 and 00 h, respectively; Fig. S. 3).

**Table 1:** Circular analyses of total moth abundance and richness (Total) and of each taxon separately

	Variables	Mean vector (*α*)	Length of mean vector (*r*)	Circular standard deviation (SD)	Rayleigh test (*Z*)	Rayleigh test of uniformity (*P*)
Total	Abundance	82.284° (22 h)	0.064	134.285°	5.687	0.003
	Richness	129.961° (23 h)	0.101	122.689°	3.325	0.036
Arctiinae	Abundance	66.908° (22 h)	0.058	136.69°	4.447	0.012
	Richness	114.737° (23 h)	0.06	135.841°	1.025	0.359
Sphingidae	Abundance	152.893° (00 h)	0.384	79.319°	5.738	0.003
	Richness	158.724° (00 h)	0.358	82.111°	3.591	0.026
Saturniidae	Abundance	114.384° (23 h)	0.466	70.81°	5.428	0.004
	Richness	127.336° (23 h)	0.514	66.087°	3.965	0.016

**Fig. 3:**
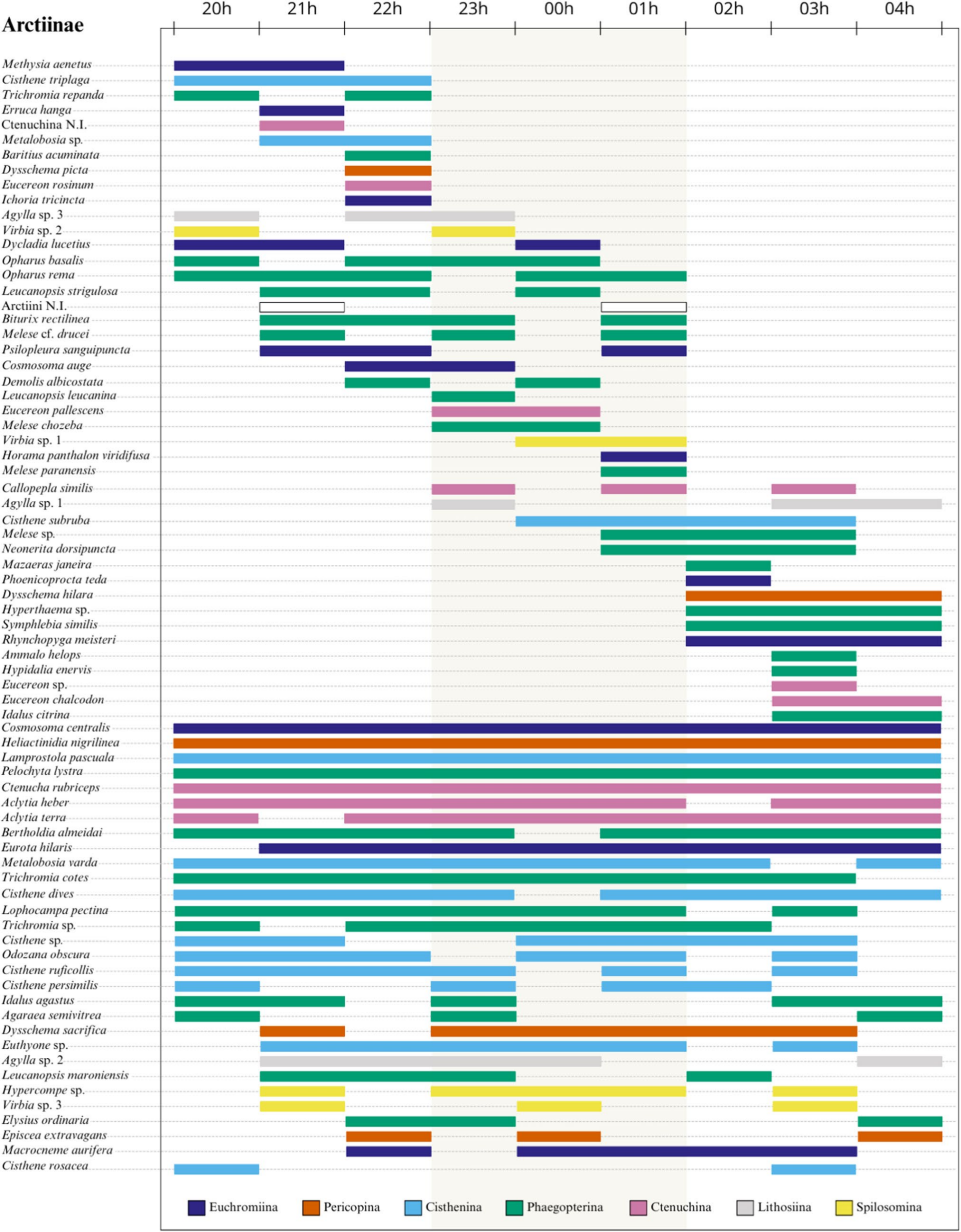
Hours of activity of Arctiinae moths sampled in December 2023 in the Coxilha do Fogo locality, Canguçu, RS, Brazil. Colors correspond to subtribes. The central region of the figure (marked in darker color) delimits the middle of the night period (23 to 01 h). Species are ordered by temporal niche breadth, from top to bottom, in the following order: (1) active only in the beggining of the night; (2) active in the beggining and middle; (3) exclusive to middle; (4) active in middle and end; (5) exclusive to end; (6) active the whole night; (7) active in different periods with no clear patterns

**Fig. 4:**
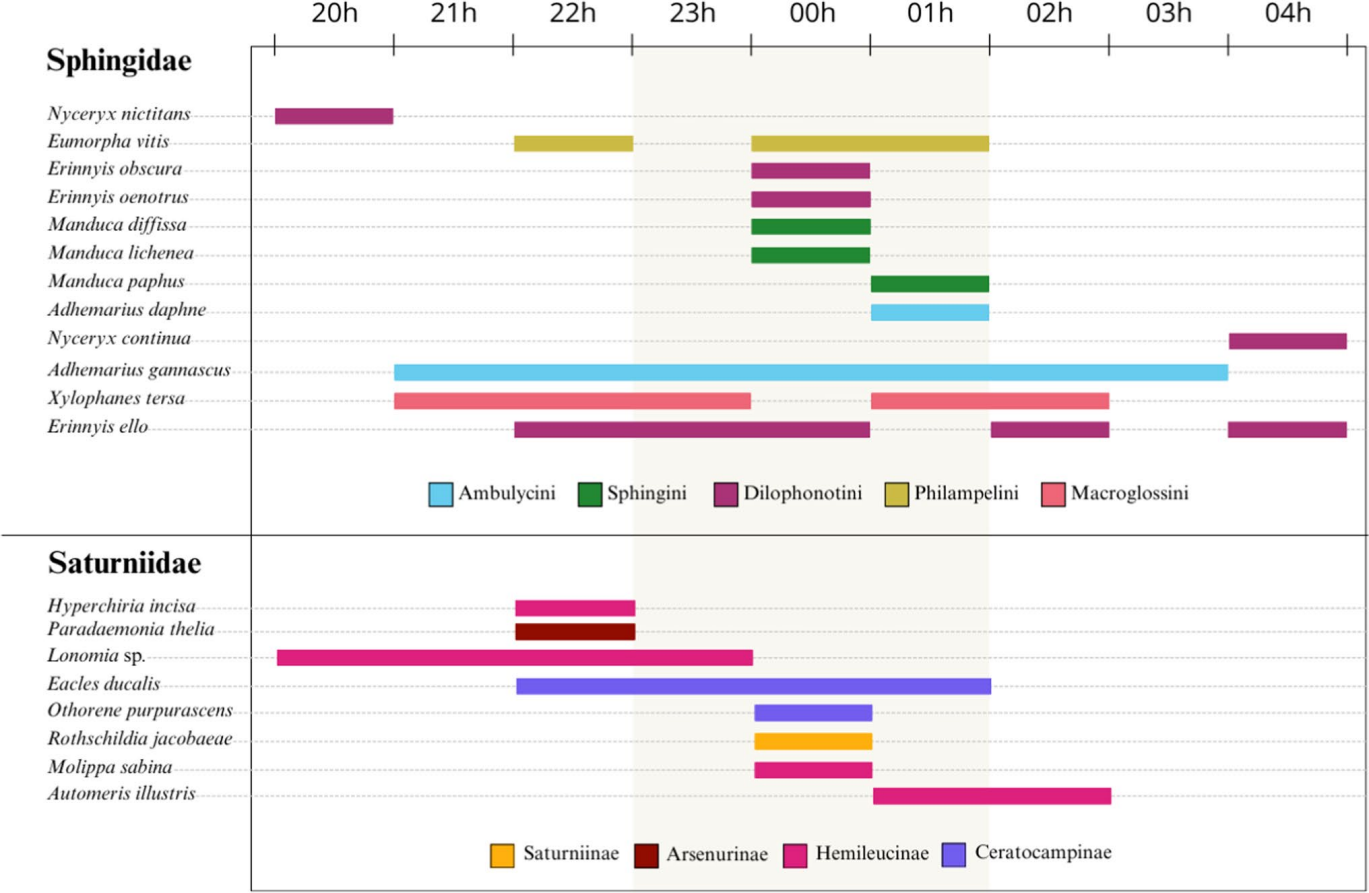
Hours of activity of Sphingidae and Saturniidae moths sampled in December 2023 in the Coxilha do Fogo locality, Canguçu, RS, Brazil. Colors correspond to subtribes. The central region of the figure (marked in darker color) delimits the middle of the night period (23 to 01 h). Species are ordered by temporal niche breadth, from top to bottom, in the following order: (1) active only in the beggining of the night; (2) active in the beggining and middle; (3) exclusive to middle; (4) active in middle and end; (5) exclusive to end; (6) active the whole night; (7) active in different periods with no clear patterns

For the community as a whole, only five species (5.32%) were active from dusk to dawn (Figs. 3 and 4). Thirteen species (13.83%) were found only in the early evening (20–22 h), 15 species (15.96%) were sampled only in the middle of the night period (23–01 h), and 12 species (12.77%) occurred only in the late evening (02–04 h). The majority (28.72%) of the species remained active for only 1 h during the night (Fig. S. 2). The second most frequent strategy was being active for 3 h (20.21%), followed by 2 h (14.89%).

### Climatic Influence

Relative humidity showed an average increasing trend as the night progressed, with the lowest recorded humidity being 66% and the highest 94% (Table S. 1). Temperature, on the other hand, showed little variation over the six nights of sampling (Table S. 1). The highest temperature recorded was 26.2°C and the lowest was 17.1°C; the largest fluctuation in one night was from 26.2 to 22.1°C, and the lowest from 23.9 to 23.2°C. Wind speed did not show a clear increasing or decreasing trend over time, although higher values were recorded at the beginning of the night and lower values at the end. Precipitation occurred only in the early hours of one of the sampling nights (20 and 21 h), being completely absent on all other nights/hours.

The abundance and richness of both the Total sample and Arctiinae were negatively correlated with wind speed and precipitation (Table 2, Table S. 3, Figure S. 4). For both, neither abundance nor richness was affected by temperature, relative humidity, or hours after sunset. Saturniid abundance was positively related to temperature and relative humidity, and negatively affected by wind speed and hours after sunset; the same results were observed for Saturniid richness (Table 2, Table S. 3, Figure S. 4). For Sphingidae, a positive correlation with temperature was found for both richness and abundance, although the explanatory power of the models was low (5 and 6%) (Table 2, Table S. 3).

**Table 2:** Influential variables for abundance and richness of each taxon, according to Generalized Linear Model (GLM) analysis. *p < 0.1; **p < 0.05; ***p < 0.0

Táxon	Dependent variables	Variables selected by GLM	*R*^2^
Total	Abundance	Wind (−0.588**) Precipitation (−1.823**)	0.209
	Richness	Wind (−0.369*) Precipitation (−1.382***)	0.210
Arctiinae	Abundance	Wind (−0.591**) Precipitation (−1.796***)	0.205
	Richness	Wind (−0.364*) Precipitation (−1.324***)	0.206
Sphingidae	Abundance	Temperature (0.186*)	0.062
	Richness	Temperature (0.155*)	0.049
Saturniidae	Abundance	Temperature (1.147***) Humidity (0.494***) Wind (−2.175**) Hours after sunset (−0.639***)	0.420
	Richness	Temperature (0.888***) Humidity (0.394**) Wind (−2.042*) Hours after sunset (−0.499**)	0.343

The variable *hours after sunset* was significant for 10 of the 13 species analyzed individually (7 species with decreasing abundance as night passes and 3 with an increase) (Table S. 4, Figure S. 5). The variable was non-influential only for *Cosmosoma centralis*, *Eurata hilaris* (Euchromiina), and *Bertholdia almeidai* (Phaegopterina). The second most present variable in the individual species GLM analyses was *humidity* (4 species affected positively by the increase and 3 affected negatively), followed by *temperature* (4 species affected positively by the increase and 2 affected negatively) and *wind* (6 species affected negatively by the increase) (Table S. 4, Figure S. 5). The species with the most significant environmental variables included in their final models were *Rhynchopyga meisteri* (*R*^2^ = 0.933) and *Lamprostola pascuala* (*R*^2^ = 0.564) (Table S. 4).

## Discussion

In just six nights of sampling, we recorded approximately 22.5% of arctiines, 14.3% of sphingids, and 6.4% of saturniids from Rio Grande do Sul State (Ferro and Teston 2009; Nunes 2006; Specht et al. 2008a). Most of the moths sampled were arctiines. This is explained by the taxon being much more diverse (approximately 1400 Brazilian species [Ferro and Diniz 2010]) than Saturniidae (488 Brazilian species [Carneiro et al. 2024]) and Sphingidae (196 Brazilian species [Haxaire and Mielke 2019]). Our data supports the first hypothesis, with different taxa having different abundance/richness concentrations throughout the night. Also, we confirm our second hypothesis, with Arctiinae peaking earlier in the night than Sphingidae and Saturniidae. The third hypothesis was partially corroborated, with smaller-sized species (Arctiinae) being more affected by variables that affect flight mechanics, while bigger ones (Sphingidae and Saturniidae) were more correlated with factors that affect flight physiology. As shall be discussed in sequence, we highlight that in our study: (1) richness and abundance varied across hours and among taxa; (2) climatic factors had contrasting effects between bigger- and smaller-sized groups and even within species of the same group; (3) a full-night sampling is necessary for optimal faunal inventories.

### Climate Effects

Given the high correlation between abundance and richness, the climatic factors that determined the abundance of each taxon also determined their respective richness. Furthermore, as expected, the variables that explained the temporal patterns of abundance and richness were the same for the entire moth assemblage and for Arctiinae, as the subfamily represents over 95% of the individuals sampled.

The activity of smaller moths (Arctiinae) was most influenced by wind speed and precipitation, which negatively impact flight. Rain and wind can prevent flight and decrease the abundance of both moths and other winged insects (Botero-Garcés & Isaacs 2004; Lawson and Rands 2019; McGeachie 1989; Poulsen 1996). Wind can directly affect foraging patterns (Hennessy et al. 2020) and interfere in pheromone communication between males and females (Cardé 2016). Similarly to wind, the impact of precipitation is much more significant on small insects than on larger ones, destabilizing flight and damaging wing and body structures (Ortega-Jimenez and Dudley 2012). The accumulation of rainwater on the body can also affect thermoregulation, increasing the individual’s mass and, consequently, the energy cost of flight (Lawson and Rands 2019). The environmental noise of rain can also potentially interfere with the interpretation of audio, visual, and olfactory signals (Lawson and Rands 2019).

For larger moths (Saturniidae and Sphingidae), climatic variables that influence flight physiology (air temperature and relative humidity) were more important. Sphingidae was the taxon least affected by climatic variables, being influenced only by temperature (positively correlated). Camargo et al. (2016a) also observed that the activity of Amazonian sphingids was related to temperature. Moth’s flight is an energetically expensive activity, and oxygen consumption during flight grows exponentially with the increase in the individual’s mass (Bartholomew and Casey 1978). When taking flight, many species need to reach specific body temperatures, which are achieved by warming their muscles through vibrations before flight (Dotterweich 1928; Heinrich 1993). The heat generated during flight by muscular activity depends on the individual’s mass, wingbeat frequency, and wing loading; in other words, larger, more robust moths produce more heat and lose it slowly, while smaller species heat and cool their bodies at faster rates (Bartholomew and Heinrich 1973). From this perspective, although larger species could maintain body temperature for longer, smaller species would have a much lower energy cost to take flight, since larger individuals consume more oxygen to warm their muscles (Bartholomew and Casey 1978). The correlation of saturniids and sphingids with temperature may, therefore, be directly related to their physiological needs for flight, since an environment with higher temperatures would make muscle warming easier. Furthermore, the hovering flight of sphingids is a high-energy-demanding behavior (Bartholomew and Casey 1978), and any factors that affect muscle warming might be relevant for sphingid activity. The sensitivity of saturniids to temperature and humidity may also be related to the fact that they do not feed as adults, and survive on stored energy acquired as larva (Braga and Diniz 2018; Janzen 1984). Although there are exceptions (e.g., Kafley and Smetacek 2020), the lack of fluid intake in adult saturniids makes them especially susceptible to desiccation, a factor that likely explains the family’s sensitivity to relative humidity and association with rainy seasons in other Brazilian biomes (Barcellos et al. 2022; Braga and Diniz 2018). We emphasize that, given the small sample size resulting from the low natural abundance of Sphingidae and Saturniidae in the area, interpretations of the physiology and ecology of these families naturally require further studies and robust empirical evidence for the subsequent determination of behavioral adaptations to the Biome climate.

It is suggested that moths with tympanic organs/ears have slower and less costly flights and, consequently, may have lower thoracic temperatures (Rydell and Lancaster 2000). Moths without tympanic organs tend to have faster and more erratic flights than those who have it, probably due to the need to avoid predation by bats (Lewis et al. 1993). A higher wing loading is also observed in several moths without tympanic organs, a factor likely associated with higher thoracic temperatures during flight (Rydell and Lancaster 2000). Arctiines do not respond erratically to bats and possess both ears and sound defense organs (Conner et al. 2009; Dowdy and Conner 2019). In this context, by having both acoustic monitoring and defense mechanisms, it is possible that Arctiinae moths have less costly flights and require a lower body temperature to fly. These factors would explain the lack of correlation with temperature across the family as a whole. Furthermore, the volatile climate of the Southern Hemisphere, due to its proximity to large ocean masses, directly impacts the cold resistance strategies of a variety of insects (Sinclair et al. 2003). The high abundance of Arctiinae in the sample (95%) and the lack of correlation with temperature may be indicative of a set of factors that make the physiology of species in this subfamily well adapted to the Pampa environment, where a rapid response to favorable weather moments is extremely beneficial.

The effects of climatic variables also differ among species within the same clade. Some, such as the saturniid *Lonomia* sp., are tolerant to a wide range of climatic conditions. However, some species are highly climate-sensitive, having been observed foraging only within a specific range of climatic conditions. Furthermore, species within the same clade may respond differently to a given climatic variable. In Arctiinae, for example, a positive association with temperature was observed in *Eurata hilaris*, while *Heliactinidia nigrilinea* showed a negative correlation. This contrasting response could be explained by a range of factors, such as hostplant associations, different reproductive behavior, and/or morphological particularities. *H. nigrilinea* is a slender-bodied species with wide delicate wings, possibly being more vulnerable to desiccation in higher temperatures. Meanwhile, *E. hilaris* benefited from higher temperatures but was mainly absent in the beginning of the night, when the climate was generally warmer; this suggests that there are other factors determining the behavior of the species, besides climate. We were able to document remarkable cases such as *Rhynchopyga meisteri*, a very small wasp-mimic moth that occurred only in the last hours of the night (peaking exponentially at 04 h) (Table S. 2) and was negatively affected by high temperatures. *R. meisteri* is also active during the day, and the strong association with pre-sunrise hours could be an adaptation that aims to start the day-foraging earlier. Although our data permit different interpretations, the lack of ecological information about each species severely limits any possible explanations. In a climate change scenario, studies evaluating the effects of climate on activity schedules, population dynamics, interactions, and species development are increasingly important and urgent.

### Temporal Structure of the Community

Moth activity patterns varied throughout the night. Arctiines followed a previously observed trend of occurring in a narrow temporal window, of no more than 3 h (Moreno et al. 2021; Scherrer et al. 2013; Teston 2021). Higher abundance and richness of Arctiinae in the early evening are documented for the Amazon (Teston 2021) and the Brazilian Cerrado (Moreno et al. 2021; Scherrer et al. 2013). However, richness in the Amazon and Cerrado was concentrated in the early evening, whereas in our study, there was no significant concentration of richness at any time during the night despite the concentration of abundance at 22 h.

The concentration of Saturniidae richness and abundance at 23 h and their complete absence later in the night contrasted with that observed for Amazonian saturniids, where abundance and richness are constant throughout the whole night (Lamarre et al. 2015). The lack of adult feeding in saturniids is compatible with a reduced flight period, since the adult’s main objectives are mating and breeding. Flight activity in saturniids is strongly related to mate searching, and peaks in male activity have been recorded coinciding with pheromone release by females (Toliver et al. 1979). A higher release of pheromones at a specific time may be driven by several factors, including a lower presence of predators or moments of higher fecundity. Although the open fields of the Pampa may favor the aerial dispersal of pheromones by wind, our analysis did not support a positive association of Saturniidae with strong winds. At the same time, the open grassland vegetation provides little cover, leaving individuals more exposed to predators. These vegetational characteristics may be a key factor causing the reduced active period of saturniids in the study area.

The activity pattern of Sphingidae found in this study is similar to that observed in sphingids from the Malaysian rainforest, where an abundance symmetry occurs toward midnight (Beck and Linsenmair 2006). However, this pattern differed from that recorded in the Brazilian Atlantic Forest and the Amazon rainforest of French Guiana, where sphingids have a higher abundance and richness in early hours, gradually decreasing throughout the night (Lamarre et al. 2015; Machado 2014). This pattern is also recorded in the Brazilian Caatinga, where abundance is highest in the early evening, but richness remains stable (Costa 2020). In the Brazilian Amazon, however, sphingid abundance is documented as occurring in increasing peaks, with a constant richness throughout the night (Camargo et al. 2016a). This data indicates that the activity times of a given taxon can be heterogeneous between and within each biome, and may be associated with several factors such as conservation status and type of vegetation, food availability, competition, interactions with natural enemies, climate, and nighttime duration. During summer, nighttime in the Pampa is shorter than in other Brazilian biomes due to its location at higher latitudes, a factor that results in summers with longer days and shorter nights (Mills 2008). Consequently, the nighttime window is narrowed, and the duration of optimal conditions for different moth species may be reduced or reorganized compared to environments at lower latitudes. Furthermore, taxa with different feeding habits may also exhibit contrasting activity patterns. Camargo et al. (2016b) found that sphingids have a tendency to have broader temporal niches of activity than saturniids, this being caused by sphingid feeding and foraging habits.

Our data shows that the Pampa moth fauna is highly represented by smaller species, occurring in low abundances and in very narrow temporal windows of activity. If sampling only occurred until midnight, we would have lost over 54% of the individuals and excluded species that are active only at the end of the night. Therefore, for faunistic inventories, we suggest sampling throughout the whole night. Sampling at specific hours may however be valid for studies focused on species that occur at restricted periods (e.g., *Rhynchopyga meisteri*) or for species that are active at all hours.

The Pampa biome, despite being highly modified, has only about 3% of its territory in protected areas (Ribeiro et al. 2021; Souza et al. 2020). As the Serra do Sudeste is a priority area for conservation in Brazil (Ministério Do Meio Ambiente 2004), knowledge about species and their behaviors can serve as a basis for establishing biodiversity conservation strategies. Understanding the responses of different insect species and communities to climate, for example, is highly relevant in a scenario of global climate change and habitat alterations (see Ojija et al. 2025). Lack of knowledge about a species’ natural history can, in certain cases, lead to methodological errors or flaws in conservation strategies (Nanglu et al. 2023). Besides affecting the physiology of individuals, climate change can affect the distribution of both species and their hostplants, ultimately leading to extinction (Bellaver et al. 2022). The Arctiinae moth *Heliactinidia nigrilinea*, despite being highly abundant in our sample, was associated with moderate temperatures, being less abundant when temperatures were higher. This being said, the species would be directly affected by a general increase in average temperature.

This research contributes significantly to the knowledge of moth diversity and ecology in a biome that remains understudied. Future studies with Pampa moths may provide insights about how climate affects the biology of these species, as well as detail life traits and interspecific dynamics. Such information may broaden our understanding of the nocturnal lepidopterans of the Pampa, and lead to a greater understanding of the southern Neotropics moths as a whole.

## Supplementary Information

Below is the link to the electronic supplementary material.

ESM 1 (DOCX 119 KB)

ESM 2 (DOCX 45.2 KB)

ESM 3 (DOCX 459 KB)

ESM 4 (DOCX 367 KB)

ESM 5 (DOCX 832 KB)

ESM 6 (DOCX 16.1 KB)

ESM 7 (DOCX 22.1 KB)

ESM 8 (DOCX 14.3 KB)

ESM 9 (DOCX 13.4 KB)

## Data Availability

The data used in the analysis are available in the Supplementary Material of the article (see Table S. 2).
