# Supplementary material for: Are Full-Night Samplings Necessary? Unraveling the Hourly Structure and Climatic Responses of Three Moth Groups in a Brazilian Pampa Grassland
Source: Neotrop Entomol. 2026 Apr 29;55(1):45. doi: 10.1007/s13744-026-01394-7 (PMC13128753; doi:10.1007/s13744-026-01394-7)
Supplement: Supplementary file 1 — (DOCX 119 KB) [file 13744_2026_1394_MOESM1_ESM.docx]

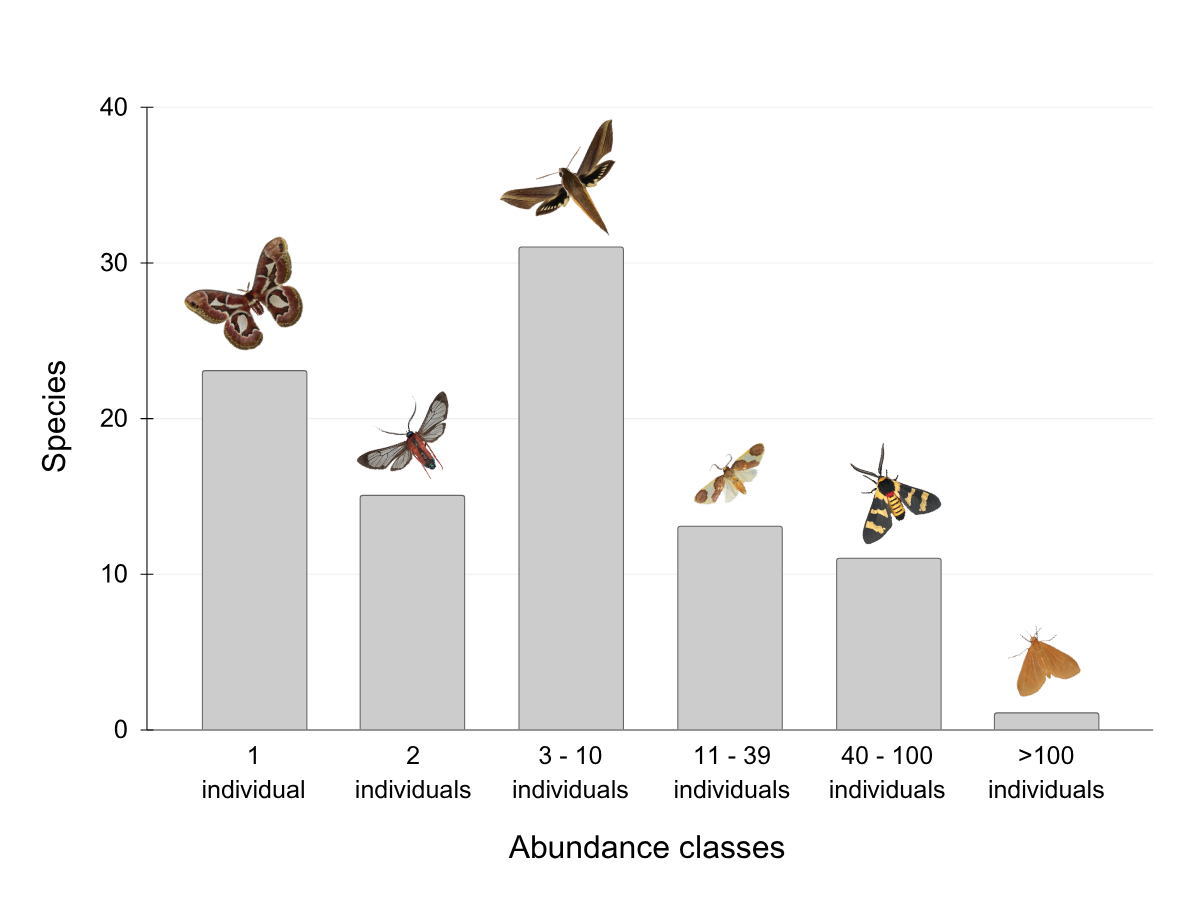


**Fig. S. 1** Number of moth species that occurred in each abundance class. Images above the bars represent examples of species from each class: *Rothschildia jacobaeae* (1 individual); *Cosmosoma auge* (2 individuals); *Xylophanes tersa* (7 individuals); *Trichromia* *cotes* (18 individuals); *Eurata hilaris* (99 individuals); *Heliactinidia nigrilinea* (235 individuals). Species out of scale
