# Supplementary material for: Are Full-Night Samplings Necessary? Unraveling the Hourly Structure and Climatic Responses of Three Moth Groups in a Brazilian Pampa Grassland
Source: Neotrop Entomol. 2026 Apr 29;55(1):45. doi: 10.1007/s13744-026-01394-7 (PMC13128753; doi:10.1007/s13744-026-01394-7)
Supplement: Supplementary file 2 — (DOCX 45.2 KB) [file 13744_2026_1394_MOESM2_ESM.docx]

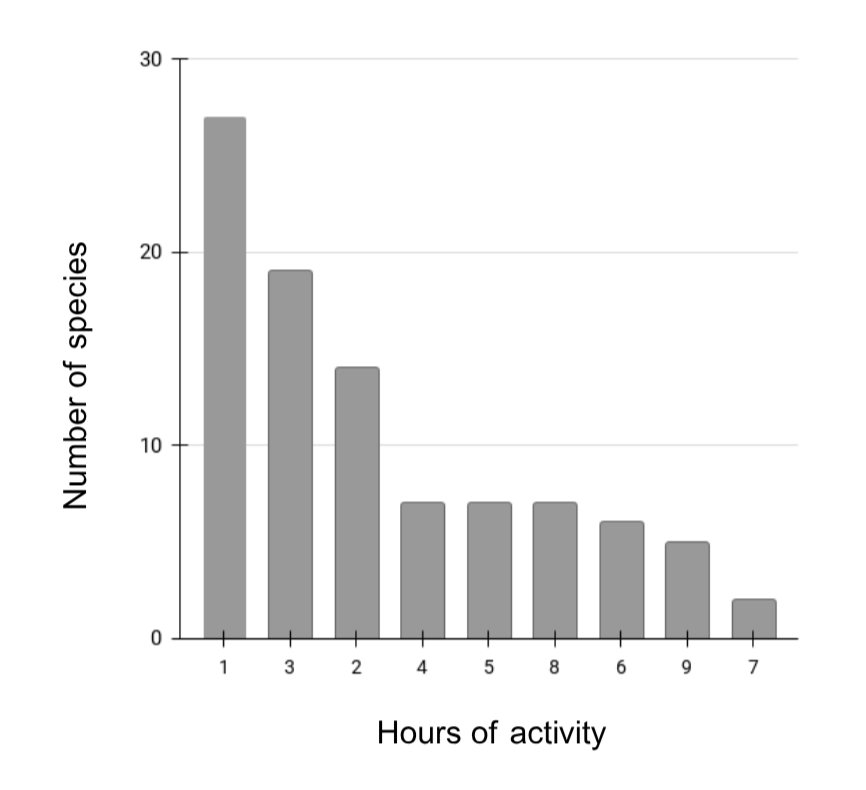


**Fig. S. 2** Relation between the number of species and the number of hours of activity, showing the most and least frequent hours of activity strategy
