# Supplementary material for: Are Full-Night Samplings Necessary? Unraveling the Hourly Structure and Climatic Responses of Three Moth Groups in a Brazilian Pampa Grassland
Source: Neotrop Entomol. 2026 Apr 29;55(1):45. doi: 10.1007/s13744-026-01394-7 (PMC13128753; doi:10.1007/s13744-026-01394-7)
Supplement: Supplementary file 3 — (DOCX 459 KB) [file 13744_2026_1394_MOESM3_ESM.docx]

**
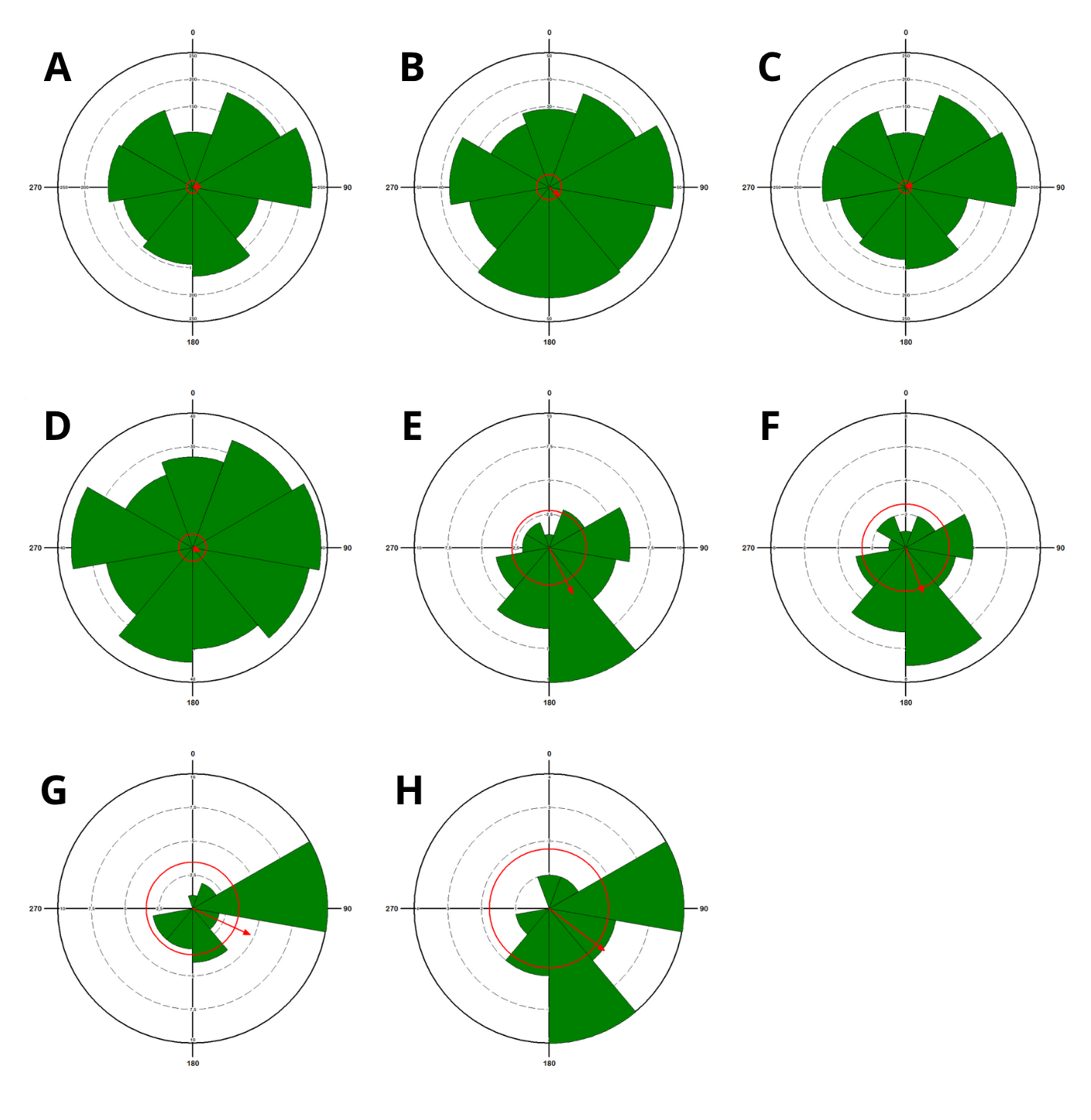
Fig. S. 3** Concentration of abundance and richness of the studied taxa. The sector centered at 0 indicates the beginning of the night period (8:00 PM). The direction and length of the red arrow indicate the average angle of data distribution (α), as well as its intensity. The red circle indicates the critical Rayleigh value. Total Abundance **(A)**; Total Richness **(B)**; Arctiinae Abundance **(C)**; Arctiinae Richness **(D)**; Sphingidae Abundance **(E)**; Sphingidae Richness **(F)**; Saturniidae Abundance **(G)** and Saturniidae Richness **(H)**
