# Supplementary material for: Are Full-Night Samplings Necessary? Unraveling the Hourly Structure and Climatic Responses of Three Moth Groups in a Brazilian Pampa Grassland
Source: Neotrop Entomol. 2026 Apr 29;55(1):45. doi: 10.1007/s13744-026-01394-7 (PMC13128753; doi:10.1007/s13744-026-01394-7)

**Figure S. 5** Significant GLM relationships between Abundance and climatic factors for each species analyzed


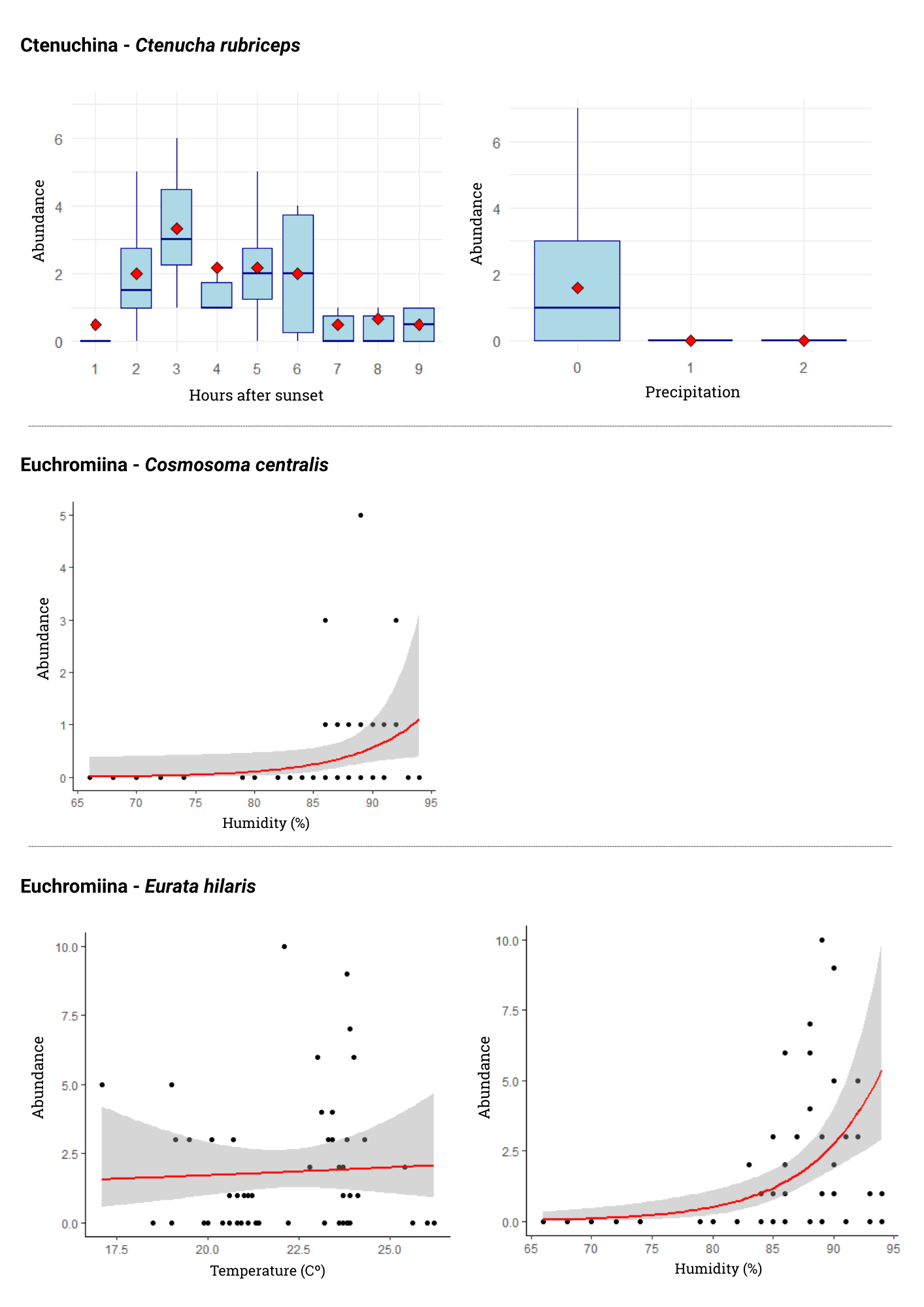


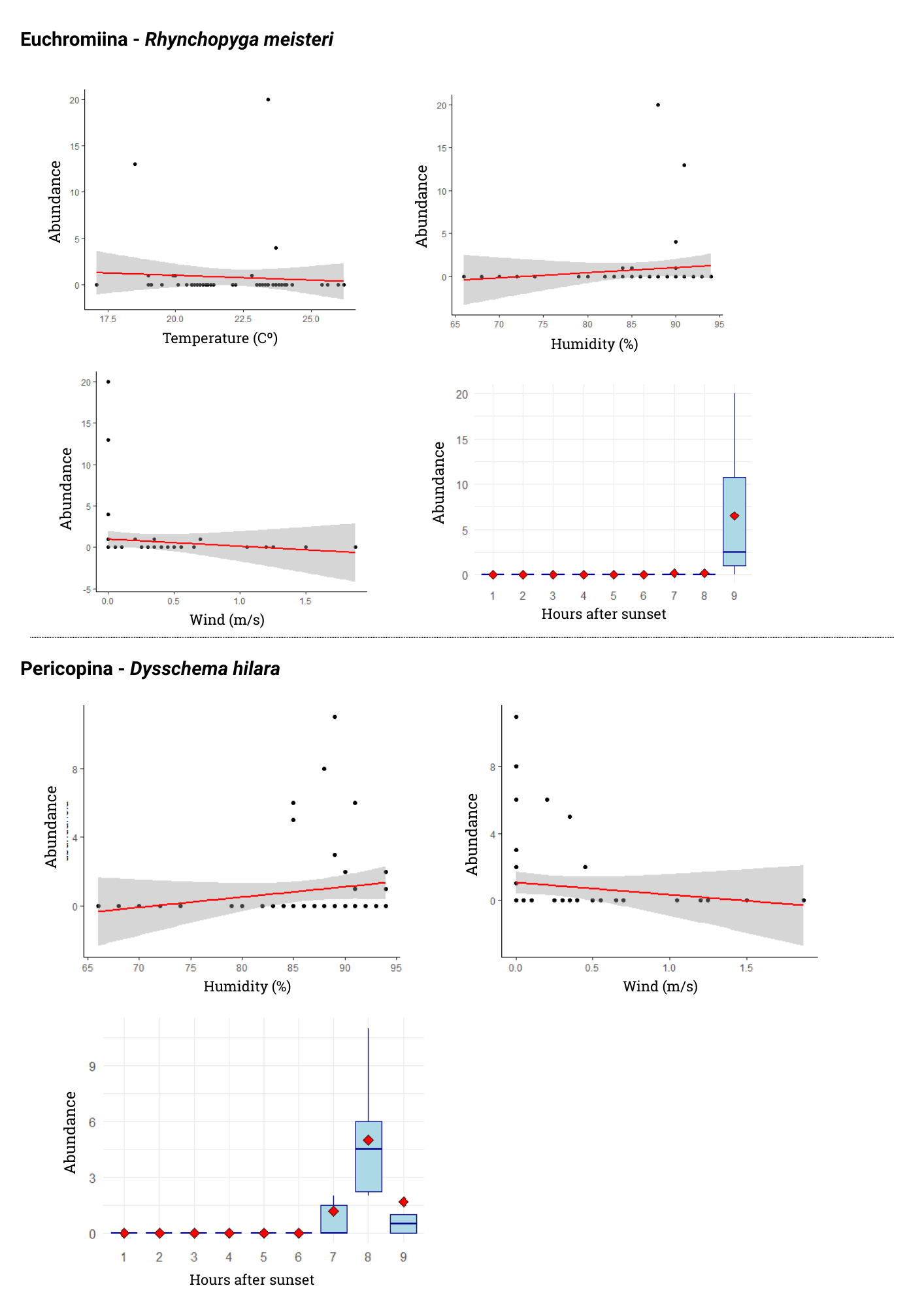


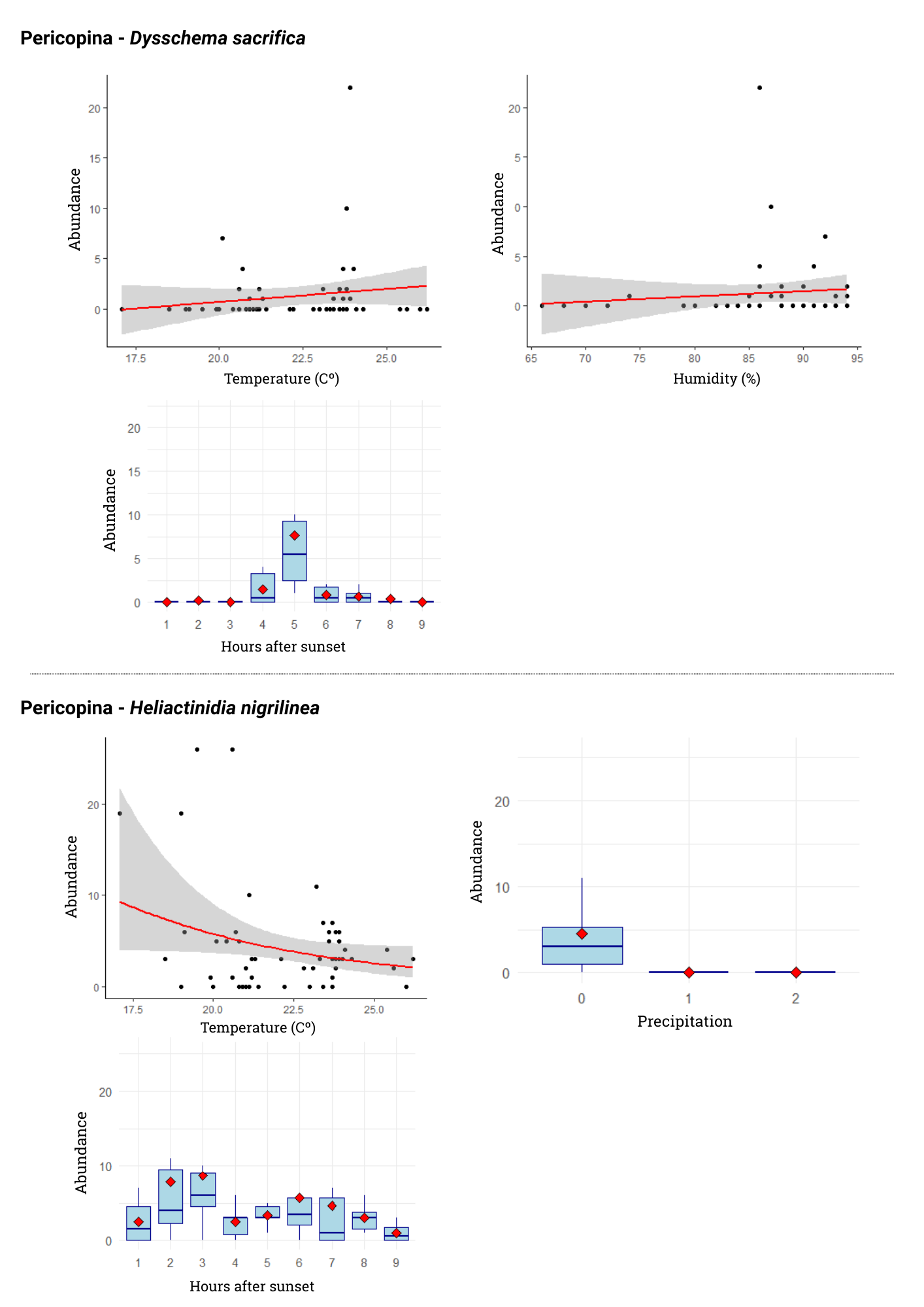


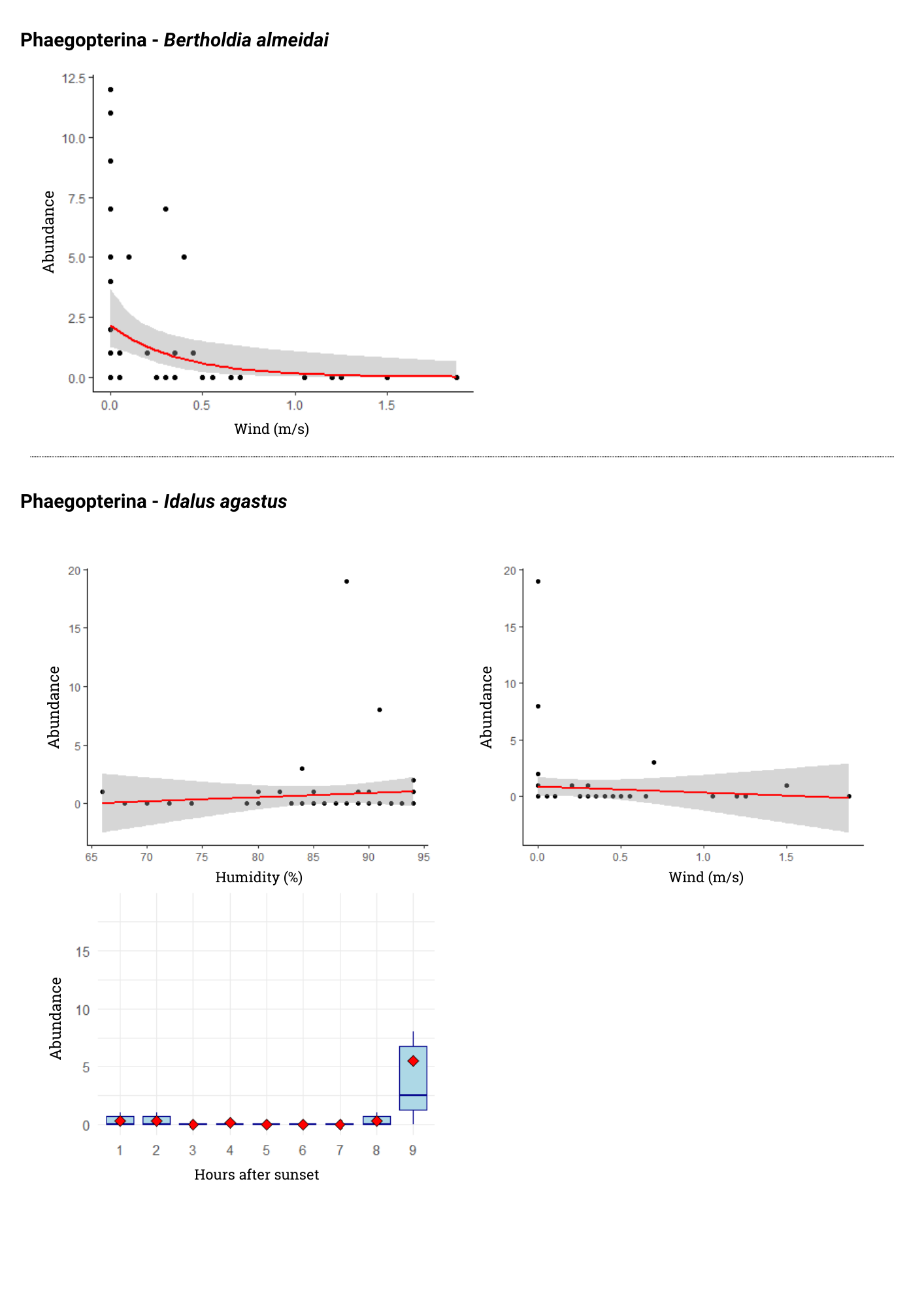


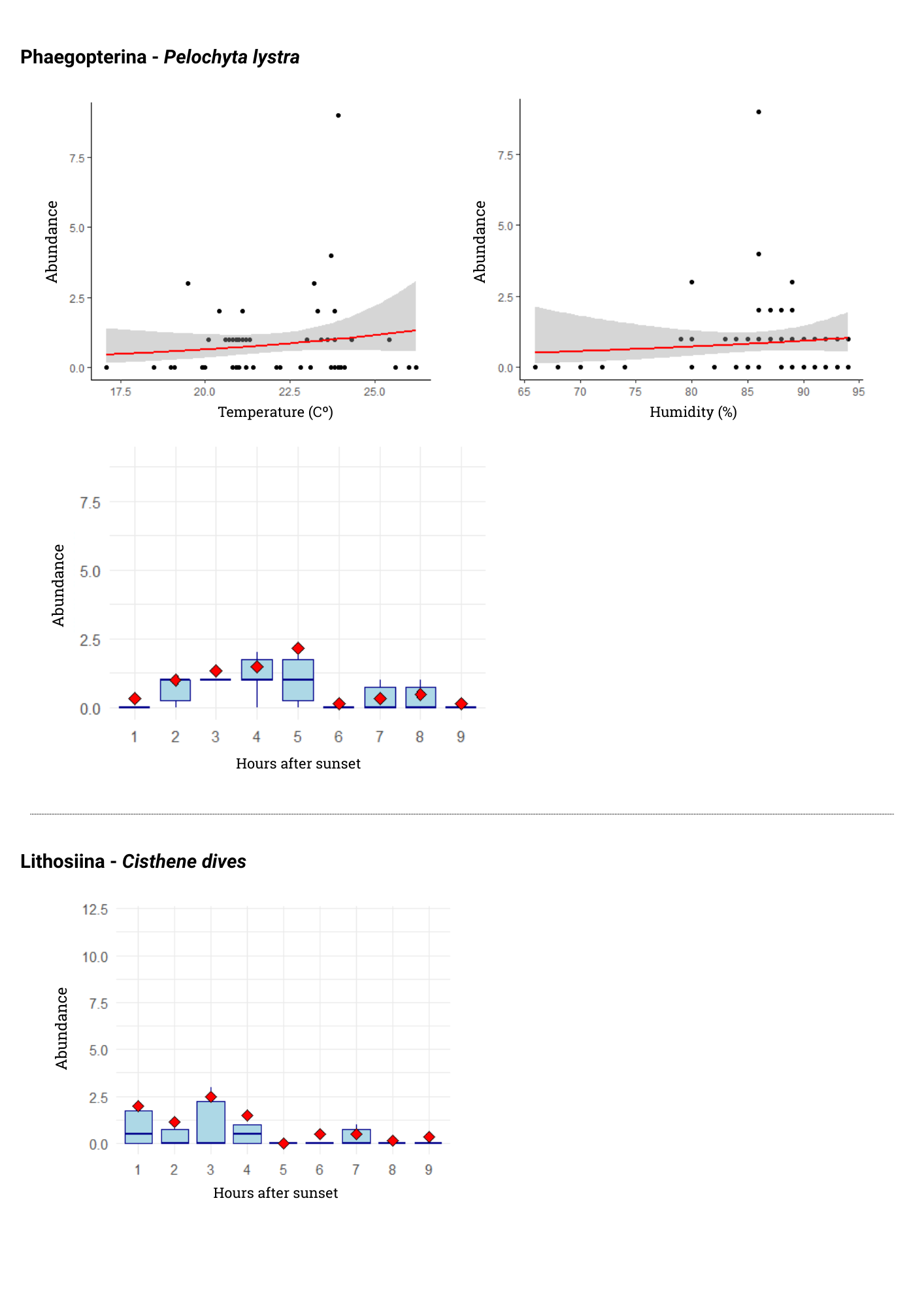

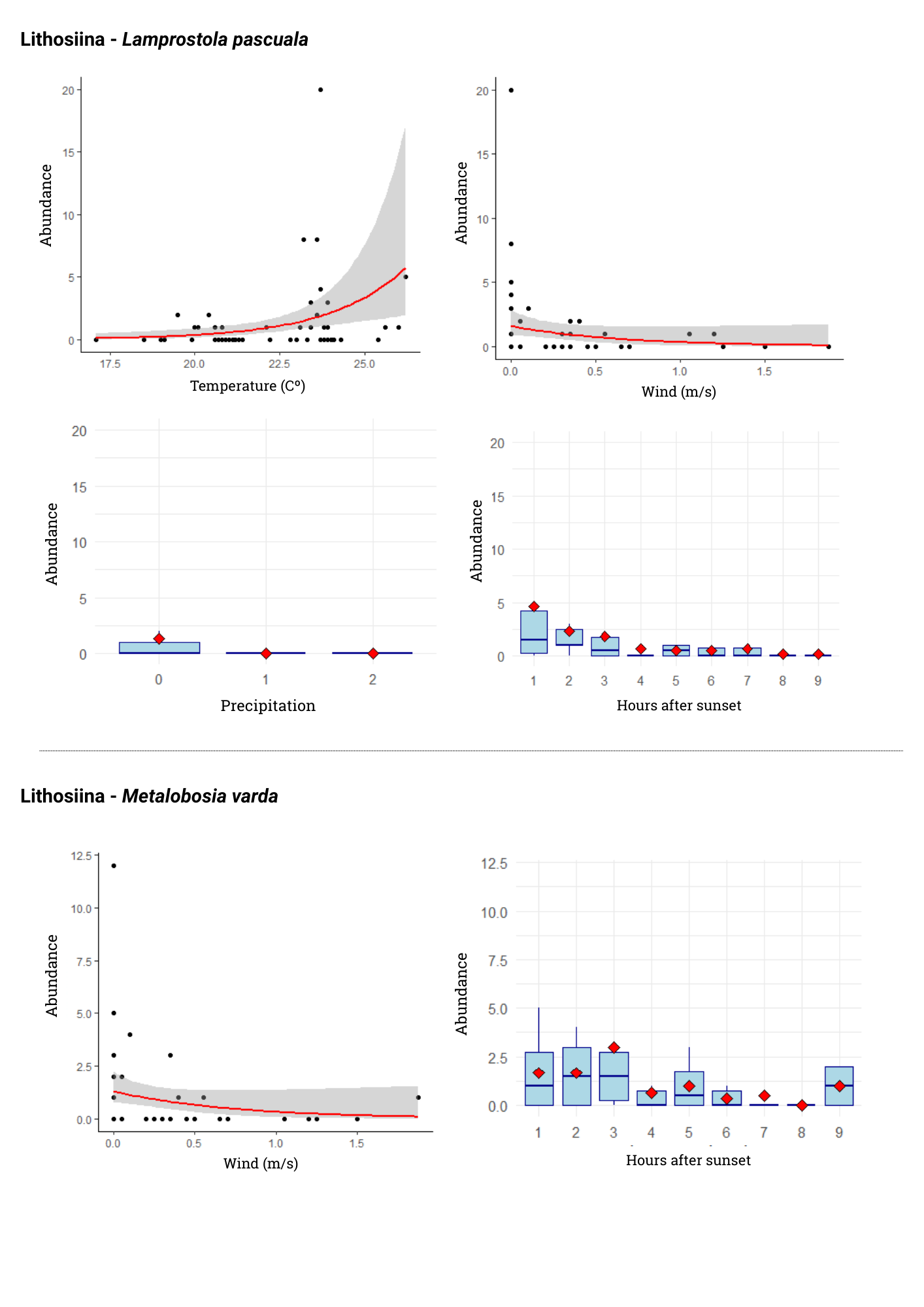

Supplement: Supplementary file 5 — (DOCX 832 KB) [file 13744_2026_1394_MOESM5_ESM.docx]
