# Supplementary material for: Are Full-Night Samplings Necessary? Unraveling the Hourly Structure and Climatic Responses of Three Moth Groups in a Brazilian Pampa Grassland
Source: Neotrop Entomol. 2026 Apr 29;55(1):45. doi: 10.1007/s13744-026-01394-7 (PMC13128753; doi:10.1007/s13744-026-01394-7)
Supplement: Supplementary file 6 — (DOCX 16.1 KB) [file 13744_2026_1394_MOESM6_ESM.docx]

**Table S. 1** Description of sampling points and weather conditions at the time of sampling. Months are in Roman numerals

| Point | Sampling date | Altitude (m) | Latitude | Longitude | Hour | Temperature (Cº) | Humidity (%) | Average wind speed (m/s) | Precipitation |
| --- | --- | --- | --- | --- | --- | --- | --- | --- | --- |
| P01 | 15-16/XII/2023 | 296 | 31° 3'16,50"S | 52°50'35,00"O | 20h | 21.4 | 82 | 0 | nule |
|  |  |  |  |  | 21h | 20.8 | 86 | 0 | nule |
|  |  |  |  |  | 22h | 21.1 | 89 | 0 | nule |
|  |  |  |  |  | 23h | 21.2 | 90 | 0 | nule |
|  |  |  |  |  | 00h | 21.3 | 93 | 0.05 | nule |
|  |  |  |  |  | 01h | 21 | 93 | 0 | nule |
|  |  |  |  |  | 02h | 20.9 | 94 | 0.3 | nule |
|  |  |  |  |  | 03h | 20.6 | 94 | 0 | nule |
|  |  |  |  |  | 04h | 20.8 | 94 | 0 | nule |
| P02 | 18-19/XII/2023 | 281 | 31° 5'13,90"S | 52°50'3,50"O | 20h | 22.2 | 68 | 0 | heavy |
|  |  |  |  |  | 21h | 20.9 | 79 | 0.25 | medium |
|  |  |  |  |  | 22h | 21 | 84 | 0.5 | nule |
|  |  |  |  |  | 23h | 21.1 | 86 | 0 | nule |
|  |  |  |  |  | 00h | 21.2 | 86 | 0 | nule |
|  |  |  |  |  | 01h | 20.8 | 85 | 0 | nule |
|  |  |  |  |  | 02h | 20 | 85 | 0.35 | nule |
|  |  |  |  |  | 03h | 19.9 | 85 | 0.2 | nule |
|  |  |  |  |  | 04h | 19 | 84 | 0.7 | nule |
| P03 | 17-18/XII/2023 | 301 | 31° 5'11,30"S | 52°49'21,60"O | 20h | 23.7 | 70 | 0 | nule |
|  |  |  |  |  | 21h | 23.2 | 80 | 0 | nule |
|  |  |  |  |  | 22h | 23.6 | 86 | 0 | nule |
|  |  |  |  |  | 23h | 23.7 | 86 | 0 | nule |
|  |  |  |  |  | 00h | 23.9 | 86 | 0 | nule |
|  |  |  |  |  | 01h | 23.6 | 86 | 0.05 | nule |
|  |  |  |  |  | 02h | 23.4 | 87 | 0 | nule |
|  |  |  |  |  | 03h | 23.3 | 89 | 0 | nule |
|  |  |  |  |  | 04h | 23.4 | 88 | 0 | nule |
| P04 | 16-17/XII/2023 | 280 | 31° 5'23,90"S | 52°50'43,60"O | 20h | 26 | 72 | 1.2 | nule |
|  |  |  |  |  | 21h | 25.6 | 80 | 1.05 | nule |
|  |  |  |  |  | 22h | 25.4 | 83 | 1.25 | nule |
|  |  |  |  |  | 23h | 24.3 | 85 | 1.5 | nule |
|  |  |  |  |  | 00h | 24 | 86 | 0.65 | nule |
|  |  |  |  |  | 01h | 23.9 | 88 | 0 | nule |
|  |  |  |  |  | 02h | 23.8 | 90 | 0.35 | nule |
|  |  |  |  |  | 03h | 24.1 | 90 | 0.45 | nule |
|  |  |  |  |  | 04h | 23.7 | 90 | 0 | nule |
| P05 | 13-14/XII/2023 | 283 | 31° 5'24,90"S | 52°51'27,40"O | 20h | 20.4 | 88 | 0.35 | nule |
|  |  |  |  |  | 21h | 20.6 | 89 | 0.3 | nule |
|  |  |  |  |  | 22h | 19.5 | 89 | 0.4 | nule |
|  |  |  |  |  | 23h | 20.7 | 91 | 1.875 | nule |
|  |  |  |  |  | 00h | 20.1 | 92 | 0.55 | nule |
|  |  |  |  |  | 01h | 19 | 92 | 0 | nule |
|  |  |  |  |  | 02h | 17.1 | 90 | 0 | nule |
|  |  |  |  |  | 03h | 19.1 | 91 | 0 | nule |
|  |  |  |  |  | 04h | 18.5 | 91 | 0 | nule |
| P06 | 14-15/XII/2023 | 266 | 31° 5'34,60"S | 52°51'44,40"O | 20h | 26.2 | 66 | 0 | nule |
|  |  |  |  |  | 21h | 23.9 | 74 | 0.1 | nule |
|  |  |  |  |  | 22h | 23.8 | 80 | 0 | nule |
|  |  |  |  |  | 23h | 23.7 | 85 | 0 | nule |
|  |  |  |  |  | 00h | 23.8 | 87 | 0 | nule |
|  |  |  |  |  | 01h | 23.1 | 88 | 0 | nule |
|  |  |  |  |  | 02h | 23 | 88 | 0 | nule |
|  |  |  |  |  | 03h | 22.1 | 89 | 0 | nule |
|  |  |  |  |  | 04h | 22.8 | 90 | 0 | nule |
