# Supplementary material for: Are Full-Night Samplings Necessary? Unraveling the Hourly Structure and Climatic Responses of Three Moth Groups in a Brazilian Pampa Grassland
Source: Neotrop Entomol. 2026 Apr 29;55(1):45. doi: 10.1007/s13744-026-01394-7 (PMC13128753; doi:10.1007/s13744-026-01394-7)
Supplement: Supplementary file 7 — (DOCX 22.1 KB) [file 13744_2026_1394_MOESM7_ESM.docx]

**Table S. 2** List of species found in the study and their respective abundances per sampling hour. Arctiinae is divided by subtribe ([Lithosiina + Cisthenina = Lithosiini] [Spilosomina + Pericopina + Phaegopterina + Euchromiina + Ctenuchina = Arctiini]). Sphingidae is divided by tribe and Saturniidae by subfamily

|  | **Species** | **20h** | **21h** | **22h** | **23h** | **00h** | **01h** | **02h** | **03h** | **04h** | **Total** |
| --- | --- | --- | --- | --- | --- | --- | --- | --- | --- | --- | --- |
| **ARCTIINAE** |  |  |  |  |  |  |  |  |  |  |  |
| Lithosiina | *Agylla* sp. 1 | 0 | 0 | 0 | 1 | 0 | 0 | 0 | 2 | 5 | 8 |
|  | *Agylla* sp. 2 | 0 | 2 | 3 | 2 | 1 | 0 | 0 | 0 | 1 | 9 |
|  | *Agylla* sp. 3 | 1 | 0 | 3 | 1 | 0 | 0 | 0 | 0 | 0 | 5 |
| Cisthenina | *Cisthene dives* (Schaus, 1896) | 12 | 7 | 15 | 9 | 0 | 3 | 3 | 1 | 2 | 52 |
|  | *Cisthene persimilis* (Hampson, 1903) | 1 | 0 | 0 | 2 | 0 | 3 | 1 | 0 | 0 | 7 |
|  | *Cisthene rosacea* (Schaus, 1896) | 1 | 0 | 0 | 0 | 0 | 0 | 0 | 1 | 0 | 2 |
|  | *Cisthene ruficollis* (Schaus, 1896) | 3 | 1 | 1 | 4 | 0 | 3 | 0 | 1 | 0 | 13 |
|  | *Cisthene* sp*.* | 1 | 8 | 0 | 0 | 4 | 5 | 8 | 1 | 0 | 27 |
|  | *Cisthene subruba* (Schaus, 1905) | 0 | 0 | 0 | 0 | 2 | 2 | 1 | 2 | 0 | 7 |
|  | *Cisthene triplaga* (Hampson, 1905) | 1 | 1 | 1 | 0 | 0 | 0 | 0 | 0 | 0 | 3 |
|  | *Euthyone* sp. | 0 | 4 | 1 | 1 | 2 | 1 | 0 | 2 | 0 | 11 |
|  | *Lamprostola pascuala* (Schaus, 1896) | 28 | 14 | 11 | 4 | 3 | 3 | 4 | 1 | 1 | 69 |
|  | *Metalobosia* sp. | 0 | 1 | 2 | 0 | 0 | 0 | 0 | 0 | 0 | 3 |
|  | *Metalobosia varda* (Schaus, 1896) | 10 | 10 | 18 | 4 | 6 | 2 | 3 | 0 | 6 | 59 |
|  | *Odozana obscura* (Schaus, 1896) | 1 | 2 | 1 | 0 | 1 | 1 | 0 | 1 | 0 | 7 |
| Spilosomina | *Hypercompe* sp. | 0 | 4 | 0 | 1 | 2 | 1 | 0 | 2 | 0 | 10 |
|  | *Virbia* sp. 1 | 0 | 0 | 0 | 0 | 1 | 1 | 0 | 0 | 0 | 2 |
|  | *Virbia* sp. 2 | 1 | 0 | 0 | 1 | 0 | 0 | 0 | 0 | 0 | 2 |
|  | *Virbia* sp. 3 | 0 | 1 | 0 | 0 | 1 | 0 | 0 | 2 | 0 | 4 |
| Pericopina | *Dysschema hilara* (Weymer, 1895) | 0 | 0 | 0 | 0 | 0 | 0 | 7 | 30 | 10 | 47 |
|  | *Dysschema picta* (Guérin-Méneville, [1844]) | 0 | 0 | 1 | 0 | 0 | 0 | 0 | 0 | 0 | 1 |
|  | *Dysschema sacrifica* (Hübner, [1831]) | 0 | 1 | 0 | 9 | 46 | 5 | 4 | 2 | 0 | 67 |
|  | *Episcea extravagans* Warren, 1901 | 0 | 0 | 1 | 0 | 1 | 0 | 0 | 0 | 1 | 3 |
|  | *Heliactinidia nigrilinea* (Walker, 1856) | 15 | 47 | 52 | 15 | 20 | 34 | 28 | 18 | 6 | 235 |
| Phaegopterina | *Agaraea semivitrea* (Rothschild, 1909) | 0 | 0 | 2 | 1 | 0 | 0 | 0 | 0 | 1 | 4 |
|  | *Ammalo helops* (Cramer, [1776]) | 0 | 0 | 0 | 0 | 0 | 0 | 0 | 1 | 0 | 1 |
|  | Arctiini N.I. | 0 | 1 | 0 | 0 | 0 | 1 | 0 | 0 | 0 | 2 |
|  | *Baritius acuminata* (Walker, 1856) | 0 | 0 | 1 | 0 | 0 | 0 | 0 | 0 | 0 | 1 |
|  | *Bertholdia almeidai* Travassos, 1950 | 3 | 18 | 10 | 4 | 0 | 2 | 8 | 24 | 16 | 85 |
|  | *Biturix rectilinea* (Burmeister, 1878) | 0 | 1 | 1 | 2 | 0 | 1 | 0 | 0 | 0 | 5 |
|  | *Demolis albicostata* Hampson, 1901 | 0 | 0 | 1 | 0 | 1 | 0 | 0 | 0 | 0 | 2 |
|  | *Elysius ordinaria* (Schaus, 1894) | 0 | 0 | 1 | 1 | 0 | 0 | 0 | 0 | 1 | 3 |
|  | *Hyperthaema* sp. | 0 | 0 | 0 | 0 | 0 | 0 | 1 | 8 | 4 | 13 |
|  | *Hypidalia enervis* (Schaus, 1894) | 0 | 0 | 0 | 0 | 0 | 0 | 0 | 2 | 0 | 2 |
|  | *Idalus agastus* Dyar, 1910 | 2 | 2 | 0 | 1 | 0 | 0 | 0 | 2 | 33 | 40 |
|  | *Idalus citrina* Druce, 1890 | 0 | 0 | 0 | 0 | 0 | 0 | 0 | 4 | 2 | 6 |
|  | *Leucanopsis leucanina* (Felder & Rogenhofer, 1874) | 0 | 0 | 0 | 1 | 0 | 0 | 0 | 0 | 0 | 1 |
|  | *Leucanopsis maroniensis* (Schaus, 1905) | 0 | 2 | 1 | 1 | 0 | 0 | 1 | 0 | 0 | 5 |
|  | *Leucanopsis strigulosa* (Walker, 1855) | 0 | 16 | 12 | 0 | 1 | 0 | 0 | 0 | 0 | 29 |
|  | *Lophocampa pectina* (Schaus, 1896) | 1 | 1 | 6 | 2 | 3 | 4 | 0 | 1 | 0 | 18 |
|  | *Mazaeras janeira* (Schaus, 1892) | 0 | 0 | 0 | 0 | 0 | 0 | 1 | 0 | 0 | 1 |
|  | *Melese cf. drucei* Rothschild, 1909 | 0 | 1 | 0 | 2 | 0 | 1 | 0 | 0 | 0 | 4 |
|  | *Melese chozeba* (Druce, 1884) | 0 | 0 | 0 | 1 | 1 | 0 | 0 | 0 | 0 | 2 |
|  | *Melese* *paranensis* Dognin, 1911 | 0 | 0 | 0 | 0 | 0 | 1 | 0 | 0 | 0 | 1 |
|  | *Melese* sp. | 0 | 0 | 0 | 0 | 0 | 2 | 4 | 6 | 0 | 12 |
|  | *Neonerita dorsipuncta* Hampson, 1901 | 0 | 0 | 0 | 0 | 0 | 1 | 1 | 1 | 0 | 3 |
|  | *Opharus basalis* Walker, 1856 | 2 | 0 | 3 | 2 | 1 | 0 | 0 | 0 | 0 | 8 |
|  | *Opharus rema* (Dognin, 1891) | 1 | 1 | 3 | 0 | 1 | 1 | 0 | 0 | 0 | 7 |
|  | *Pelochyta lystra* (Druce, 1884) | 2 | 6 | 8 | 9 | 13 | 1 | 2 | 3 | 1 | 45 |
|  | *Symphlebia similis* (Rothschild, 1917) | 0 | 0 | 0 | 0 | 0 | 0 | 1 | 5 | 3 | 9 |
|  | *Trichromia cotes* (Druce, 1896) | 1 | 1 | 3 | 3 | 2 | 5 | 2 | 1 | 0 | 18 |
|  | *Trichromia repanda* (Walker, 1855) | 1 | 0 | 1 | 0 | 0 | 0 | 0 | 0 | 0 | 2 |
|  | *Trichromia* sp. | 1 | 0 | 1 | 2 | 1 | 2 | 1 | 0 | 0 | 8 |
| Euchromiina | *Cosmosoma auge* (Linnaeus, 1767) | 0 | 0 | 1 | 1 | 0 | 0 | 0 | 0 | 0 | 2 |
|  | *Cosmosoma centralis* (Walker, 1854) | 1 | 5 | 1 | 2 | 2 | 6 | 1 | 2 | 1 | 21 |
|  | *Dycladia lucetius* (Stoll, 1781) | 1 | 1 | 0 | 0 | 1 | 0 | 0 | 0 | 0 | 3 |
|  | *Erruca hanga* (Herrich-Schäffer, [1854]) | 0 | 1 | 0 | 0 | 0 | 0 | 0 | 0 | 0 | 1 |
|  | *Eurata hilaris* Zerny, 1937 | 0 | 1 | 6 | 9 | 14 | 20 | 23 | 17 | 9 | 99 |
|  | *Horama panthalon viridifusa* (Schaus, 1904) | 0 | 0 | 0 | 0 | 0 | 1 | 0 | 0 | 0 | 1 |
|  | *Ichoria tricincta* (Herrich-Schäffer, 1855) | 0 | 0 | 1 | 0 | 0 | 0 | 0 | 0 | 0 | 1 |
|  | *Macrocneme aurifera* Hampson, 1914 | 0 | 0 | 1 | 0 | 2 | 4 | 12 | 1 | 0 | 20 |
|  | *Phoenicoprocta teda* (Walker, 1854) | 0 | 0 | 0 | 0 | 0 | 0 | 1 | 0 | 0 | 1 |
|  | *Psilopleura sanguipuncta* Hampson, 1898 | 0 | 1 | 2 | 0 | 0 | 1 | 0 | 0 | 0 | 4 |
|  | *Rhynchopyga meisteri* (Berg, 1883) | 0 | 0 | 0 | 0 | 0 | 0 | 1 | 1 | 39 | 41 |
| Ctenuchina | *Aclytia heber* (Cramer, 1780) | 3 | 6 | 4 | 2 | 2 | 2 | 0 | 2 | 3 | 24 |
|  | *Aclytia terra* Schaus, 1896 | 3 | 0 | 3 | 2 | 2 | 1 | 1 | 1 | 1 | 14 |
|  | *Callopepla similis* (Heylaerts, 1890) | 0 | 0 | 0 | 1 | 0 | 1 | 0 | 1 | 0 | 3 |
|  | *Ctenucha rubriceps* Walker, 1854 | 3 | 12 | 20 | 13 | 13 | 12 | 3 | 4 | 3 | 83 |
|  | Ctenuchina sp. | 0 | 1 | 0 | 0 | 0 | 0 | 0 | 0 | 0 | 1 |
|  | *Eucereon chalcodon* Druce, 1893 | 0 | 0 | 0 | 0 | 0 | 0 | 0 | 1 | 1 | 2 |
|  | *Eucereon pallescens* Rothschild, 1912 | 0 | 0 | 0 | 1 | 1 | 0 | 0 | 0 | 0 | 2 |
|  | *Eucereon rosinum* (Walker, 1854) | 0 | 0 | 2 | 0 | 0 | 0 | 0 | 0 | 0 | 2 |
|  | *Eucereon* sp. | 0 | 0 | 0 | 0 | 0 | 0 | 0 | 1 | 0 | 1 |
|  | *Methysia aenetus* (Schaus, 1896) | 1 | 1 | 0 | 0 | 0 | 0 | 0 | 0 | 0 | 2 |
|  | **Abundance- Arctiinae** | 101 | 182 | 205 | 117 | 151 | 134 | 123 | 155 | 150 | **1318** |
|  | **Richness - Arctiinae** | 27 | 34 | 38 | 35 | 30 | 34 | 26 | 36 | 23 | **74** |
| **SPHINGIDAE** |  |  |  |  |  |  |  |  |  |  |  |
| Ambulycini | *Adhemarius daphne* (Boisduval, [1875]) | 0 | 0 | 0 | 0 | 0 | 1 | 0 | 0 | 0 | 1 |
|  | *Adhemarius gannascus* (Stoll, 1790) | 0 | 2 | 3 | 3 | 2 | 1 | 1 | 2 | 0 | 14 |
| Dilophonotini | *Erinnyis ello* (Linnaeus, 1758) | 0 | 0 | 1 | 1 | 2 | 0 | 1 | 0 | 1 | 6 |
|  | *Erinnyis obscura* (Fabricius, 1775) | 0 | 0 | 0 | 0 | 1 | 0 | 0 | 0 | 0 | 1 |
|  | *Erinnyis oenotrus* (Cramer, 1780) | 0 | 0 | 0 | 0 | 1 | 0 | 0 | 0 | 0 | 1 |
|  | *Nyceryx continua* (Walker, 1856) | 0 | 0 | 0 | 0 | 0 | 0 | 0 | 0 | 1 | 1 |
|  | *Nyceryx nictitans* (Boisduval, [1875]) | 1 | 0 | 0 | 0 | 0 | 0 | 0 | 0 | 0 | 1 |
| Macroglossini | *Xylophanes tersa* (Linnaeus, 1771) | 0 | 1 | 1 | 1 | 0 | 2 | 2 | 0 | 0 | 7 |
| Philampelini | *Eumorpha vitis* (Linnaeus, 1758) | 0 | 0 | 1 | 0 | 2 | 1 | 0 | 0 | 0 | 4 |
| Sphingini | *Manduca diffissa* (Butler, 1871) | 0 | 0 | 0 | 0 | 1 | 0 | 0 | 0 | 0 | 1 |
|  | *Manduca lichenea* (Burmeister, 1855) | 0 | 0 | 0 | 0 | 1 | 0 | 0 | 0 | 0 | 1 |
|  | *Manduca paphus* (Cramer, 1779) | 0 | 0 | 0 | 0 | 0 | 1 | 0 | 0 | 0 | 1 |
|  | **Abundance- Sphingidae** | 1 | 3 | 6 | 5 | 10 | 6 | 4 | 2 | 2 | **39** |
|  | **Richness - Sphingidae** | 1 | 2 | 4 | 3 | 7 | 5 | 3 | 1 | 2 | **12** |
| **SATURNIIDAE** |  |  |  |  |  |  |  |  |  |  |  |
| Hemileucinae | *Automeris illustris* (Walker, 1855) | 0 | 0 | 0 | 0 | 0 | 2 | 3 | 0 | 0 | 5 |
|  | *Hyperchiria incisa* Walker, 1855 | 0 | 0 | 2 | 0 | 0 | 0 | 0 | 0 | 0 | 2 |
|  | *Lonomia* sp. | 1 | 2 | 5 | 1 | 0 | 0 | 0 | 0 | 0 | 9 |
|  | *Molippa sabina* Walker, 1855 | 0 | 0 | 0 | 0 | 1 | 0 | 0 | 0 | 0 | 1 |
| Ceratocampinae | *Eacles ducalis* (Walker, 1855) | 0 | 0 | 1 | 1 | 1 | 1 | 0 | 0 | 0 | 4 |
|  | *Othorene purpurascens* (Schaus, 1905) | 0 | 0 | 0 | 0 | 1 | 0 | 0 | 0 | 0 | 1 |
| Arsenurinae | *Paradaemonia thelia* (Jordan, 1922) | 0 | 0 | 2 | 0 | 0 | 0 | 0 | 0 | 0 | 2 |
| Saturniinae | *Rothschildia jacobaeae* (Walker, 1855) | 0 | 0 | 0 | 0 | 1 | 0 | 0 | 0 | 0 | 1 |
|  | **Abundance - Saturniidae** | 1 | 2 | 10 | 2 | 4 | 3 | 3 | 0 | 0 | **25** |
|  | **Richness - Saturniidae** | 1 | 1 | 4 | 2 | 4 | 2 | 1 | 0 | 0 | **8** |
|  | **Abundance - Total** | 103 | 187 | 221 | 124 | 165 | 143 | 130 | 157 | 152 | **1382** |
|  | **Richness - Total** | 29 | 37 | 46 | 40 | 41 | 41 | 30 | 37 | 25 | **94** |
