# Supplementary material for: Are Full-Night Samplings Necessary? Unraveling the Hourly Structure and Climatic Responses of Three Moth Groups in a Brazilian Pampa Grassland
Source: Neotrop Entomol. 2026 Apr 29;55(1):45. doi: 10.1007/s13744-026-01394-7 (PMC13128753; doi:10.1007/s13744-026-01394-7)
Supplement: Supplementary file 8 — (DOCX 14.3 KB) [file 13744_2026_1394_MOESM8_ESM.docx]

**Table S. 3** Generalized Linear Models (GLMs) overall results, showing the influential variables for abundance and richness of each taxon throughout the night. Note: *p<0.1; **p<0.05; ***p<0.01

| **Total** | *Dependent variable* | |  | **Arctiinae** | *Dependent variable* | |
| --- | --- | --- | --- | --- | --- | --- |
|  | abundance | richness |  |  | abundance | richness |
| wind | -0.588**  (-1.092; -0.085) | -0.369*  (-0.743; 0.006) |  | wind | -0.591^**^  (-1.099; -0.083) | -0.364*  (-0.735; 0.007) |
| precipitation | -1.823***  (-2.896; -0.750) | -1.382***  (-2.336; -0.428) |  | precipitation | -1.796^***^  (-2.869; -0.723) | -1.324***  (-2.265; -0.383) |
| Constant | 3.377***  (3.139; 3.615) | 2.468***  (2.297; 2.638) |  | Constant | 3.336^***^  (3.096; 3.576) | 2.379***  (2.212; 2.546) |
| R² McFadden | 0.209 | 0.21 |  | R² McFadden | 0.205 | 0.206 |
| Observations | 54 | 54 |  | Observations | 54 | 54 |
| Log Likelihood | -222.214 | -166.802 |  | Log Likelihood | -220.276 | -161.645 |
| theta | 1.810^***^  (0.360) | 4.675***  (1.319) |  | theta | 1.787^***^  (0.356) | 5.122^***^  (1.536) |
| Akaike Inf. Crit. | 450.427 | 339.604 |  | Akaike Inf. Crit. | 446.553 | 329.290 |
|  |  |  |  |  |  |  |
| **Sphingidae** | *Dependent variable* | |  | **Saturniidae** | *Dependent variable* | |
|  | abundance | richness |  |  | abundance | richness |
| temperature | 0.186*  (-0.013; 0.385) | 0.155*  (-0.015; 0.325) |  | temperature | 1.147***  (0.471; 1.824) | 0.888***  (0.238; 1.538) |
| Constant | -4.508*  (-9.032; 0.017) | -3.917**  (-7.803; -0.032) |  | humidity | 0.494***  (0.163; 0.825) | 0.394**  (0.079; 0.709) |
| R² McFadden | 0.062 | 0.049 |  | wind | -2.175**  (-4.194; -0.156) | -2.042*  (-4.364; 0.280) |
| Observations | 54 | 54 |  | hours after sunset | -0.639***  (-1.059; -0.220) | -0.499**  (-0.918; -0.079) |
| Log Likelihood | -62.601 | -58.355 |  | Constant | -66.913***  (-108.077; -25.750) | -53.044***  (-91.819; -14.269) |
| theta | 1.507 (1.067) | - |  | R² McFadden | 0.42 | 0.343 |
| Akaike Inf. Crit. | 129.202 | 120.711 |  | Observations | 54 | 54 |
|  |  |  |  | Log Likelihood | -28.656 | -26.031 |
|  |  |  |  | theta | - | - |
|  |  |  |  | Akaike Inf. Crit. | 67.312 | 62.063 |
