# Supplementary material for: Are Full-Night Samplings Necessary? Unraveling the Hourly Structure and Climatic Responses of Three Moth Groups in a Brazilian Pampa Grassland
Source: Neotrop Entomol. 2026 Apr 29;55(1):45. doi: 10.1007/s13744-026-01394-7 (PMC13128753; doi:10.1007/s13744-026-01394-7)
Supplement: Supplementary file 9 — (DOCX 13.4 KB) [file 13744_2026_1394_MOESM9_ESM.docx]

**Table S. 4** Influential variables for species abundance throughout the night according to Generalized Linear Models (GLM). Note: *p<0.1; **p<0.05; ***p<0.01

| Tribe | Subtribe | Species | Variables selected by GLM | R² |
| --- | --- | --- | --- | --- |
| Arctiini | Ctenuchina | *Ctenucha rubriceps* | hours after sunset (-0.161***)  precipitation (-31.997) | 0.157 |
|  | Euchromiina | *Cosmosoma centralis* | humidity (0.167**) | 0.131 |
|  |  | *Eurata hilaris* | temperature (0.223**)  humidity (0.222***) | 0.296 |
|  |  | *Rhynchopyga meisteri* | temperature (-0.476***)  humidity (-0.928***)  wind (-12.316***)  hours after sunset (4.047***) | 0.933 |
|  | Pericopina | *Dysschema hilara* | humidity (-0.147**)  wind (-1.881**)  hours after sunset (0.839***) | 0.513 |
|  |  | *Dysschema sacrifica* | temperature (0.614***)  humidity (0.272***)  hours after sunset (-0.267***) | 0.211 |
|  |  | *Heliactinidia nigrilinea* | temperature (-0.208***)  hours after sunset (-0.186***)  precipitation (-34.023) | 0.278 |
|  | Phaegopterina | *Bertholdia almeidai* | wind (-2.634**) | 0.138 |
|  |  | *Idalus agastus* | humidity (-0.250***)  wind (-2.200**)  hours after sunset (1.010***) | 0.482 |
|  |  | *Pelochyta lystra* | temperature (0.334**)  humidity (0.178***)  hours after sunset (-0.362***) | 0.227 |
| Lithosiini | Lithosiina | *Cisthene dives* | hours after sunset (-0.295**) | 0.126 |
|  |  | *Lamprostola pascuala* | temperature (0.294**)  wind (-2.072***)  hours after sunset (-0.407***)  precipitation (-29.857) | 0.564 |
|  |  | *Metalobosia varda* | wind (-1.709**)  hours after sunset (-0.232***) | 0.203 |
